# Inflammatory metabolic profile of South African patients with prostate cancer

**DOI:** 10.1186/s40170-021-00265-6

**Published:** 2021-08-03

**Authors:** Stefano Cacciatore, Martha Wium, Cristina Licari, Aderonke Ajayi-Smith, Lorenzo Masieri, Chanelle Anderson, Azola Samkele Salukazana, Lisa Kaestner, Marco Carini, Giuseppina M. Carbone, Carlo V. Catapano, Massimo Loda, Towia A. Libermann, Luiz F. Zerbini

**Affiliations:** 1 Cancer Genomics Group, International Centre for Genetic Engineering and Biotechnology, Cape Town, South Africa; 2 Institute for Reproductive and Developmental Biology, Imperial College, London, UK; 3 Magnetic Resonance Center (CERM), University of Florence, Sesto Fiorentino, Italy; 4 Department of Urology, Clinica Urologica I, Azienda Ospedaliera Careggi, University of Florence, Florence, Italy; 5 Pediatric Urology Unit, Meyer Children Hospital, University of Florence, Florence, Italy; 6 Division of Urology, University of Cape Town, Groote Schuur Hospital, Cape Town, South Africa; 7 Institute of Oncology Research (IOR), Università della Svizzera italiana, Bellinzona, Switzerland; 8 Swiss Institute of Bioinformatics (SIB), Lausanne, Switzerland; 9 Department of Oncology, Faculty of Biology and Medicine, University of Lausanne, Lausanne, Switzerland; 10 Department of Oncologic Pathology, Dana-Farber Cancer Institute, Boston, MA USA; 11 Department of Pathology and Laboratory Medicine, Weill Cornell Medicine, New York, NY USA; 12 Harvard Medical School, MA Boston, USA; 13 BIDMC Genomics, Proteomics, Bioinformatics and Systems Biology Center, Beth Israel Deaconess Medical Center, MA Boston, USA

**Keywords:** Metabolomics, NMR spectroscopy, GlycA, GlycB, Histidine, Prostate cancer, Africa

## Abstract

**Background:**

Men with African ancestry are more likely to develop aggressive prostate cancer (PCa) and to die from this disease. The study of PCa in the South African population represents an opportunity for biomedical research due to the high prevalence of aggressive PCa. While inflammation is known to play a significant role in PCa progression, its association with tumor stage in populations of African descent has not been explored in detail. Identification of new metabolic biomarkers of inflammation may improve diagnosis of patients with aggressive PCa.

**Methods:**

Plasma samples were profiled from 41 South African men with PCa using nuclear magnetic resonance (NMR) spectroscopy. A total of 41 features, including metabolites, lipid classes, total protein, and the inflammatory NMR markers, GlycA, and GlycB, were quantified from each NMR spectrum. The Bruker’s B.I.-LISA protocols were used to characterize 114 parameters related to the lipoproteins. The unsupervised KODAMA method was used to stratify the patients of our cohort based on their metabolic profile.

**Results:**

We found that the plasma of patients with very high risk, aggressive PCa and high level of C-reactive protein have a peculiar metabolic phenotype (metabotype) characterized by extremely high levels of GlycA and GlycB. The inflammatory processes linked to the higher level of GlycA and GlycB are characterized by a deep change of the plasma metabolome that may be used to improve the stratification of patients with PCa. We also identified a not previously known relationship between high values of VLDL and low level of GlycB in a different metabotype of patients characterized by lower-risk PCa.

**Conclusions:**

For the first time, a portrait of the metabolic changes in African men with PCa has been delineated indicating a strong association between inflammation and metabolic profiles. Our findings indicate how the metabolic profile could be used to identify those patients with high level of inflammation, characterized by aggressive PCa and short life expectancy. Integrating a metabolomic analysis as a tool for patient stratification could be important for opening the door to the development of new therapies. Further investigations are needed to understand the prevalence of an inflammatory metabotype in patients with aggressive PCa.

**Supplementary Information:**

The online version contains supplementary material available at 10.1186/s40170-021-00265-6.

## Background

Prostate cancer (PCa) is the second most frequent cancer diagnosis made in men. Progression to metastasis and the emergence of therapeutically resistant disease lead PCa to be the fifth cause of death worldwide [1]. Inflammation seems to play a key role in PCa development and progression with a significant impact on processes in the tumor microenvironment that facilitate progression to advanced disease [2].

While the link between inflammation and PCa is well-studied, its biological effects are often ignored in biomarker studies [3], including metabolomic studies. The metabolic changes driven by inflammation are not identified in the majority of studies on cancer. This lack leads to a bias in the understanding of mechanisms involved in the metabolic changes. Since inflammation has a strong impact on the human metabolome [3], metabolic analysis may illuminate systemic metabolic consequences of inflammation and provide novel targets for intervention. In particular, nuclear magnetic resonance (NMR) spectroscopy is a powerful technique when applied to the high-throughput analysis of biofluids such as blood [4], which can be collected with minimal impact on the participant. NMR-based metabolomics is a straightforward and useful method for the qualitative and quantitative analysis of a wide range of components in blood samples, including low-molecular weight metabolites and lipoproteins (different for size and composition) [5]. Moreover, NMR spectroscopy allows the detection in plasma of signals arising from the glycosylation of circulating acute-phase proteins (APPs), such as α1-antichymotrypsin, haptoglobin-1, α1-antitrypsin, transferrin, and α1-acid glycoprotein [6].

The carbohydrate portions of glycoproteins containing N-acetylglucosamine and N-acetylgalactosamine (hereinafter referred to as GlycA) and N-acetylneuraminic acid (a.k.a., sialic acid; hereinafter referred to as GlycB) moieties are visible as two distinct NMR signals. GlycA and GlycB levels have been associated with common markers of inflammation such as C-reactive protein (CRP), fibrinogen, interleukin (IL)-6, tumor necrosis factor-alpha, lipoprotein-associated phospholipase A_2_, and serum amyloid A [7–10]. Similar to CRP, GlycA and GlycB are markers of chronic inflammation [9, 11, 12]; despite the similarity, GlycA and CRP likely capture different aspects of the inflammatory response [9]. CRP is an “early” APP and the proteins that contribute the most to the GlycA and GlycB signal rise later in the acute phase response [13]. In response to acute and chronic inflammatory stimuli, both the concentrations of APPs and their glycan structures are modified [13]. While in acute inflammation, the increase in biantennary structures [14, 15] and the content of fucosylated glycans [14] reach the maximum value within the first few days; in chronic inflammation, the glycan structures evolve in their complexity due to the activities of different intracellular and secreted hepatic glycosyltransferases during the inflammatory cascade [16]. The number of studies linking the association of one or multiple single-nucleotide polymorphisms in inflammation-related pathways to PCa risk has greatly increased [17, 18]. Polymorphisms in immune-related genes could at least partially explain the different incidence and mortality of PCa in African men [19]. This hypothesis could be also suggested by the fact that African American (AA) men, compared to European American (EA) men, show an increased incidence of inflammation in biopsy specimens [20] and increased expression of immune-related genes in tumor tissues [21]. A distinct genomic landscape of PCa and immune-related genes associated with specific ethnic groups may lead to different metabolic adaptations that permit tumor cells to proliferate. Lately, more effort has been put on exploring the genetic factors contributing to PCa in men of African ancestry [22–26].

Among Africans, the South African population is a unique blend of African and non-African ancestry [27]. Currently, the major ethnolinguistic groups in South Africa are Black South-Eastern Bantu-speakers, an admixed population referred to as Colored [28], Whites of European origin, and a population group originating from the Indian sub-continent [27]. In a recent study, all patients who underwent a prostate biopsy from July 2008 to July 2014 at the Groote Schuur Hospital (Cape Town, South Africa) were recorded [29]. Among all patients diagnosed with PCa, 41% and 21% were classified as high- and very high-risk PCa (NCCN classification), respectively. Although the percentage of clinically advanced cases is already impressive if compared to American or European studies, the relative percentage of cases with very high-risk PCa is even higher (33%) if we consider only the Black population. The ethnic diversity and the high prevalence of aggressive PCa in the South African population represents an opportunity for biomedical research [30]. Despite the higher prevalence of aggressive PCa, the number of studies in African populations is still limited.

This study, to our knowledge, is the first to define the metabolic profile of PCa in men from Africa. Here, we used NMR spectroscopy to quantify a panel of 41 signals, including metabolites, lipid groups, proteins, and the inflammatory markers GlycA and GlycB. Moreover, we used an advanced lipoprotein test based on NMR spectroscopy to characterize the lipoprotein subclasses in each sample. For the first time, we report the relationship between these inflammatory biomarkers (i.e., GlycA and GlycB) and the most aggressive PCa cases. We provide a clear snapshot of the metabolic alterations during the inflammatory process in PCa paving the way to a better understanding of the metabolic changes occurring in PCa in men of African descent that may extend to other ethnicities as well.

## Methods

### Patients

Participants were recruited from the Urological clinics of Groote Schuur, Eerste Rivier, and New Somerset Hospitals in Cape Town, South Africa. Patients scheduled to undergo transurethral resection of the prostate or prostatectomy were enrolled. The diagnosis of PCa was confirmed by histopathologic examinations. The protocol (*HREC454/2012*) was approved by the Human Research Ethics Committee of the Faculty of Health Science, University of Cape Town, South Africa. Written consent was obtained from all the participants before 5 mL of blood was collected in Vacuette® EDTA tube by medical staff. Blood plasma was separated by centrifugation (1000×*g* for 10 min at 4 °C) and stored at – 80 °C.

### NMR sample preparation and analysis

Plasma samples were thawed at room temperature. An aliquot of 350 μL of a phosphate sodium buffer (70 mM Na_2_HPO_4_; 20% (v/v) ^2^H_2_O; 6.1 mM NaN_3_; 4.6 mM sodium 3-trimethylsilyl [2,2,3,3-^2^H_4_]-propionate; pH 7.4) was added to 350 μL of each sample. The mixture was homogenized by vortexing for 30 s, before 600 μL of this mixture was transferred into a 5-mm NMR tube for analysis.

For each plasma sample, one-dimensional ^1^H-NMR spectra were acquired on a Bruker 600 MHz spectrometer (Bruker BioSpin) operating at 600.13 MHz proton Larmor frequency and equipped with a 5 mm PATXI 1H-13C-15N and 2H-decoupling probe including a *z*-axis gradient coil, an automatic tuning-matching and an automatic and refrigerated sample changer (SampleJet). A BTO 2000 thermocouple was used at the level of approximately 0.1 K on the sample to stabilize the temperature. Before starting measurements, samples were kept inside the NMR probe head for at least 5 minutes to equilibrate temperature at 310 K. The standard Nuclear Overhauser Effect SpectroscopY (NOESY) presat pulse sequence (noesygppr1d.comp; Bruker BioSpin) was used to detect both signals of small metabolites and high-molecular weight macromolecules. Parameters of the experiment were 32 scans, 98304 data points, a spectral width of 18028.846 Hz, an acquisition time of 2.73 s, a relaxation delay of 4 s and a mixing time of 0.01 s. Transformed spectra were automatically corrected for phase and baseline distortions using Topspin 3.2 (Bruker BioSpin) and then automatically calibrated to the anomeric proton signal of α-glucose at 5.24 ppm.

### Molecular profiling and lipoprotein quantification

Lipoprotein parameters were estimated on NOESY spectra according to Bruker’s B.I.-LISA protocols (Bruker IVDr Lipoprotein subclass analysis) [31]. Information related to the main very low-density lipoprotein (VLDL), low-density lipoprotein (LDL), intermediate-density lipoprotein (IDL), and high-density lipoprotein (HDL) classes and to their subclasses were extrapolated. In detail, information was extracted of five VLDL subclasses (from VLDL-1 to VLDL-5), six LDL sub-classes (from LDL-1 to LDL-6), and four HDL-subclasses (HDL-1 to HDL-4) sorted according to increasing density and decreasing size.

For each class and subclass, calculated data consist of concentrations of lipids, i.e., cholesterol, free cholesterol, phospholipids, and triglycerides. Instead, concentrations of apolipoproteins Apo-A1 and Apo-A2 were estimated for HDL class and each relative subclass, while Apo-B concentrations are calculated for VLDL, IDL classes, and all LDL subclasses.

Identification of signals was undertaken using the SBASE database in Amix (v3.9.11; Bruker BioSpin, Germany) or available assignments in the literature. The peaks of the identified metabolites were fitted by a combination of a local baseline and Voigt functions based on the multiplicity of the NMR signal. GlycA and GlycB signals were quantified by integrating, respectively, the areas between 2.005 and 2.054 ppm and between 2.086 and 2.054 ppm above a local baseline aimed to remove the signal of the lipoproteins. Fitting methods to quantify GlycA and GlycB signals were not used due to their heterogeneity and due to the impossibility to completely distinguish them from the lipoprotein signal. The amide protein signals belong to plasma proteins were quantified integrating the area between 6.000 and 10.000 ppm.

### Plasma CRP quantification

The plasma concentration of CRP was determined using the Human C Reactive Protein ELISA kit (abcam, ab99995). Patient plasma was diluted 1:80,000 in 1× Assay Diluent D.

### Statistical and data analysis

Statistical analysis and graphical illustrations of the data were generated in the R (version 3.6.1) and R studio (version 1.1.456) software using scripts developed in-house.

Wilcoxon rank sum test and Kruskal-Wallis rank sum test were used to compare differences in numerical covariates (e.g., age and metabolite concentration). Fisher’s exact test was used to assess differences between categorical variables (e.g., ethnicity). Spearman’s test was used to calculate the correlation coefficient (rho) between variables. The KODAMA algorithm was used to facilitate the identification of patterns representing underlying metabolic phenotypes (metabotype) on all samples in the dataset. Dendrograms were performed using the KODAMA output and Ward linkage. Silhouette median value being used to evaluate the optimal number of clusters with the number of possible clusters varying from 2 to 10 [32]. *p* values less than 0.05 were considered to be significant. To account for multiple testing, a false discovery rate (FDR) of < 10% was applied.

Discriminant analysis of metabolic profiles was performed using partial least-squares (PLS) analysis. To assess the predictive ability of the PLS regression model, a 10-fold cross-validation was conducted as previously described [4]. The goodness of fit parameter (*R*^2^) and the predictive ability parameter (*Q*^2^) were calculated using standard definitions [33].

## Results

### South African patient cohort

Although a few studies have been performed to investigate the metabolic alterations in the blood of patients with PCa, high-risk populations are underrepresented and limited to AAs. In this study, we recruited 41 South African patients with PCa in order to generate a better understanding of the metabolic changes in the unique South African setting. The majority of patients were characterized by a unique mixed ancestry (61%) referred to as Colored; the rest were self-classified as Black (22%) and Whites (17%). We classified the aggressiveness of cancer according to the National Comprehensive Cancer Network (NCCN) classification (version 2.2020): (i) very low, low, and intermediate risk; (ii) high risk; and (iii) very high risk. The clinical and demographic features of the patients with PCa are reported in Table 1. Patients with regional or distant metastasis were classified as a separate group. Patients that received androgen-deprivation therapy (ADT), i.e., bilateral orchidectomy (BO), were considered as two distinct groups based on the evidence of castration-resistant PCa (CRPC). In our cohort, we did not observe statistically significant difference among ethnicity in term of NCCN classification in untreated patients, although, we reported an advanced clinical stage in Black men with 71% of them classified as stage T3 or T4 compared to 19% of Colored and 33% of White men. This disparity was highlighted even by the PSA level. We reported extremely high values of PSA (> 100 ng/mL) in 57% of Black compared to 9% of Colored and 33% of White. As expected, we observed a higher prevalence of diabetes and hypertension in the post-BO group.

**Table 1:** Clinical demographics of PCa patients

	Treatment-naïve					Post-BO		
Feature	Intermediate, low and very low (***n*** = 14)	High (***n*** = 7)	Very high (***n*** = 12)	Metastatic (***n*** = 1)	Total ***n*** = 34)	Non-CRPC (***n*** = 4)	CRPC (***n*** = 3)	Total (***n*** = 7)
Age (year), median [95% CI]	65 [56 77]	70 [52 90]	64 [57 86]	75 [75 75]	68 [63 74]	72 [63 73]	66 [64 70]	70 [65 72]
Ancestry, *n* (%)								
Black	1 (7.1)	1 (14.3)	4 (33.3)	1 (100.0)	7 (20.6)	1 (25.0)	1 (33.3)	2 (28.6)
Colored	10 (71.4)	5 (71.4)	6 (50.0)	0 (0.0)	21 (61.8)	2 (50.0)	1 (33.3)	3 (42.8)
Colored/Black	0 (0.0)	0 (0.0)	0 (0.0)	0 (0.0)	0 (0.0)	1 (25.0)	0 (0.0)	1 (14.3)
White	3 (21.4)	1 (14.3)	2 (16.7)	0 (0.0)	6 (17.6)	0 (0.0)	1 (33.3)	1 (14.3)
PSA (ng/mL), median [95% CI]	9 [3 19]	23 [17 31]	138 [29 3919]	>5000	21 [12 84]	4 [2 40]	332 [49 1128]	34 [4 188]
Diabetes, *n* (%)								
No	10 (71.4)	5 (71.4)	12 (100.0)	1 (100.0)	28 (82.4)	3 (75.0)	2 (66.7)	5 (71.4)
Yes	4 (28.6)	2 (28.6)	0 (0.0)	0 (0.0)	6 (17.6)	1 (25.0)	1 (33.3)	2 (28.6)
Hypertension, *n* (%)								
No	7 (50.0)	5 (71.4)	10 (83.3)	1 (100.0)	23 (67.6)	3 (75.0)	1 (33.3)	4 (57.1)
Yes	7 (50.0)	2 (28.6)	2 (16.7)	0 (0.0)	11 (32.4)	1 (25.0)	2 (66.7)	3 (42.9)
Smoker, *n* (%)								
No	10 (71.4)	6 (85.7)	9 (75.0)	1 (100.0)	26 (76.5)	4 (100.0)	1 (33.3)	5 (71.4)
Yes	4 (28.6)	1 (14.3)	3 (25.0)	0 (0.0)	8 (23.5)	0 (0.0)	2 (66.7)	2 (28.6)

### GlycA and GlycB inflammatory biomarkers

**Fig. 1:**
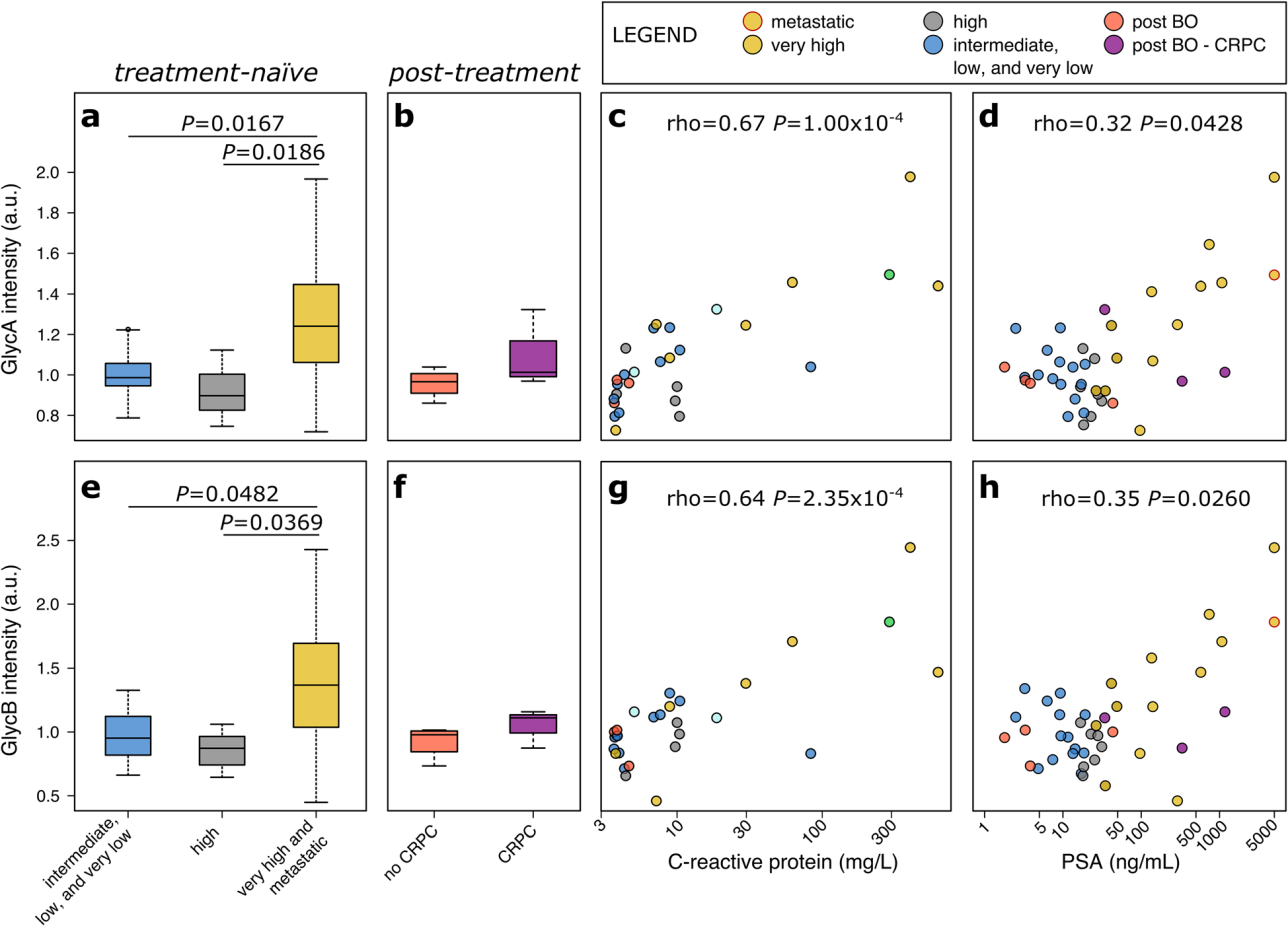
GlycA concentration in **a** treatment-naïve and **b** post-treatment. **c** Correlation between GlycA and CRP. **d** Correlation between GlycA and PSA. GlycB concentration in **e** treatment-naïve and **f** post-treatment. **g** Correlation between GlycB and CRP. **h** Correlation between GlycA and PSA

Growing evidence implicates chronic inflammation as a contributor to PCa development and progression to advanced metastatic disease [2], and as a driver of CRPC development in ADT [34, 35]. Recently, GlycA and GlycB have been identified as markers of systemic and chronic inflammation [6, 11, 12] but their association with PCa has not been described yet. Here, we used the NMR spectroscopy to quantify the signal associated with GlycA and GlycB and, for the first time, we investigated their association with the aggressiveness of PCa. We noted that the values of both markers are higher in patients with very highly aggressive PCa and metastatic PCa (Fig. 1a, e). Indeed, all patients whose GlycA and GlycB was higher than the 80th percentile were diagnosed with poorly differentiated PCa (i.e., Gleason score higher than or equal to 8). Although the limited number of patients who had BO did not allow for enough statistical power, we observed an increased value of both GlycA and GlycB in patients with CRPC (Fig. 1b, f).

Several studies have reported the association of GlycA and GlycB with key markers of cancer stratification, such as CRP [36] (Fig. 1c, g). Here, we report for the first time a statistically significant correlation with PSA (Fig. 1d, h). The correlation with PSA implies that a disparity in the GlycA and GlycB related to ethnicity should be expected in patients with PCa. Noteworthy, the three highest values of GlycA and GlycB were found in patients that identified themselves as Black.

Moreover, we quantified the amides of proteins from the NMR spectra. Albumin is the most concentrated protein in the plasma and consequently, the protein amides concentration is highly related to the albumin level. We reported a negative correlation between the NMR inflammatory marker GlycA (rho = − 0.41; *P* = 0.00747) and GlycB (rho = − 0.31; *P* = 0.0462) with protein amides. Albumin is known to be a negative APP that decreases in concentration during inflammation. This further supports our finding that the higher values of GlycA and GlycB could be associated with inflammatory process in patients with PCa.

### Metabolic stratification of PCa

**Fig. 2:**
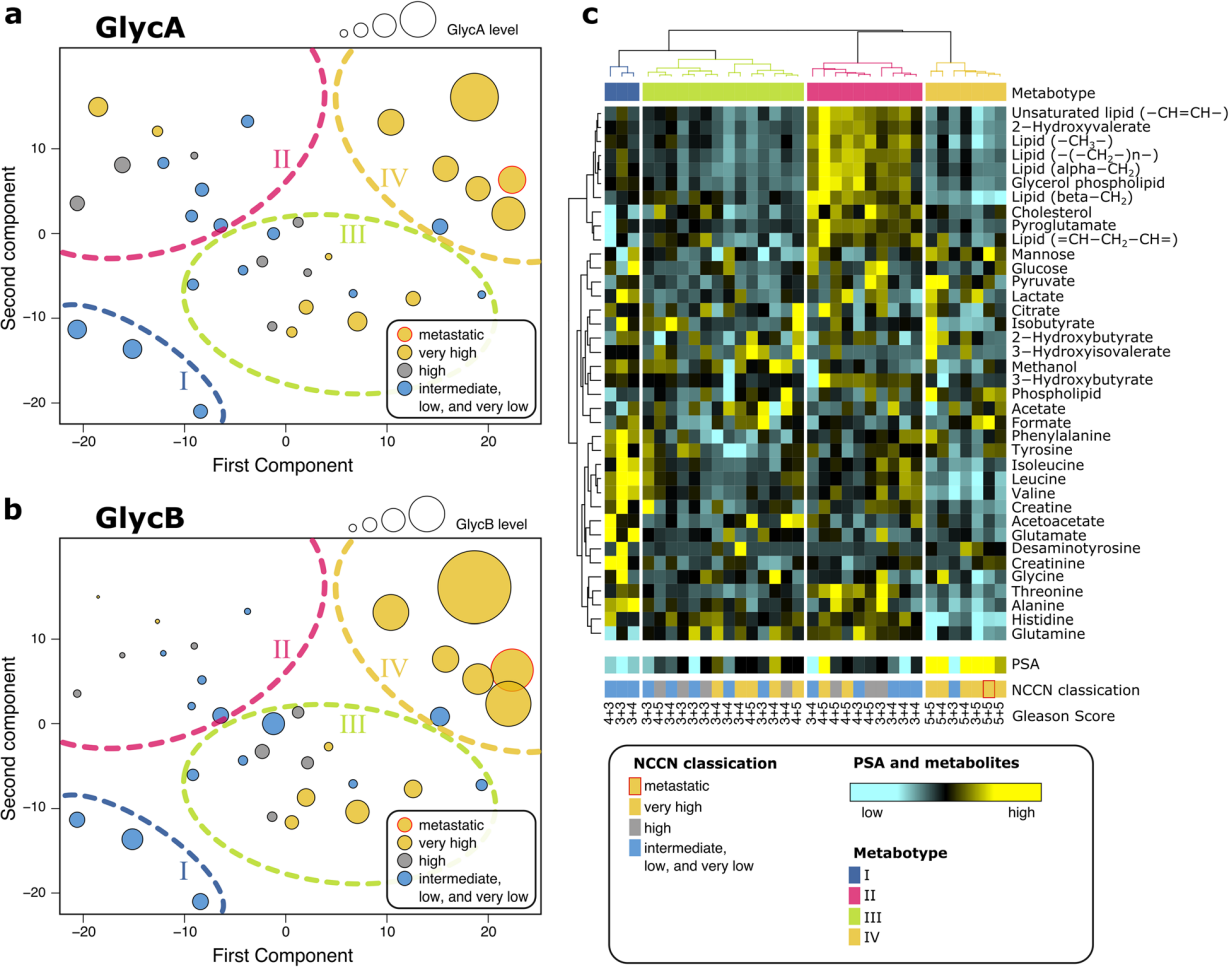
KODAMA score plot of plasma PCa samples colored according to NCCN classification. The size is proportional to **a** the GlycA intensity and **b** the GlycB intensity. **c** Heatmap of the metabolic profiles

Reprogramming of metabolism is a widely accepted hallmark of cancer development [3]; however, the metabolic changes induced by inflammation in cancer patients have not been fully characterized. Metabolomics represents an essential tool for the stratification of cancer patients into groups of patients with similar metabolic profiles that could share the same clinicopathologic condition (e.g., systemic inflammation). Here, we quantified the metabolites from each plasma sample using the data collected by the NMR experiments. In order to identify potential underlying metabolic phenotypes (a.k.a. metabotype) in patients with treatment-naïve PCa, we applied the unsupervised method KODAMA method to the quantified metabolite concentrations (GlycA, GlycB, and protein amides were not considered in this analysis). We identified four different metabotypes in the KODAMA score plot (Fig. 2a, b) using the hierarchical clustering [37] on the KODAMA scores.

We clearly observed an association between the PCa aggressiveness (based on the NCCN classification) and the metabotypes that we rank from I to IV in order of aggressiveness. When evaluating GlycB and GlycA in the 4 metabotypes, we noted different levels of GlycB in each metabotypes with the highest levels of both GlycA and GlycB in Metabotype IV. Clinical and demographic features of the metabotypes are shown in Table 2.

**Table 2:** Clinical and demographic features of the metabotypes identified thought KODAMA analysis

Feature	Metabotype I	Metabotype II	Metabotype III	Metabotype IV	***p*** value
NCCN classification, *n* (%)					1.14 × 10^−1^
Very low	1 (33.3)	0 (0.0)	1 (7.1)	0 (0.0)	
Low	0 (0.0)	1 (10.0)	1 (7.1)	1 (14.3)	
Intermediate	2 (66.7)	4 (40.0)	3 (21.4)	0 (0.0)	
High	0 (0.0)	3 (30.0)	4 (28.5)	0 (0.0)	
Very high	0 (0.0)	2 (20.0)	5 (35.7)	5 (71.4)	
Metastatic	0 (0.0)	0 (0.0)	0 (0.0)	1 (14.3)	
Gleason score, *n* (%)					9.00 × 10^−2^
3 + 3	1 (33.3)	1 (10.0)	5 (35.7)	1 (14.3)	
3 + 4	1 (33.3)	4 (40.0)	6 (42.9)	0 (0.0)	
4 + 3	1 (33.3)	1 (10.0)	0 (0.0)	0 (0.0)	
3 + 5	0 (0.0)	0 (0.0)	0 (0.0)	1 (14.3)	
4 + 5	0 (0.0)	4 (40.0)	3 (21.4)	0 (0.0)	
5 + 4	0 (0.0)	0 (0.0)	0 (0.0)	2 (28.6)	
5 + 5	0 (0.0)	0 (0.0)	0 (0.0)	3 (42.9)	
Age, median [95% CI]	71 [64 74]	68 [56 90]	65 [51 85]	74 [59 78]	9.49 × 10^−1^
Ancestry, *n*(%)					4.11 × 10^−1^
Black	1 (33.3)	1 (10.0)	2 (14.3)	3 (42.9)	
Mixed ancestry	1 (33.3)	7 (70.0)	11 (78.6)	2 (28.6)	
White	1 (33.3)	2 (20.0)	1 (7.1)	2 (28.6)	
PSA, median [95% CI]	9 [3 9]	18 [5 233]	25 [5 126]	738 [26 5000]	5.89 × 10^−3^
Diabetes, *n* (%)					8.85 × 10^−2^
No	1 (33.3)	7 (70.0)	13 (92.9)	7 (100.0)	
Yes	2 (66.7)	3 (30.0)	1 (7.1)	0 (0.0)	
Hypertension, *n* (%)					1.18 × 10^−1^
No	0 (0.0)	8 (80.0)	9 (64.3)	6 (85.7)	
Yes	3 (100.0)	2 (20.0)	5 (35.7)	1 (14.3)	
Smoker, *n* (%)					4.37 × 10^−1^
No	2 (66.7)	8 (80.0)	9 (64.3)	7 (100.0)	
Yes	1 (33.3)	2 (20.0)	5 (35.7)	0 (0.0)	

The metabolic profile of Metabotype IV is the most peculiar. Samples of Metabotype IV showed an unprecedently well-defined fingerprint that may reflect a common biologic process that drives the metabolic changes in the blood of patients with high GlycA and GlycB levels. Metabotype IV is formed almost exclusively by patients categorized as very high risk and also includes a patient with metastatic PCa (Table 2).

Noteworthy, a patient classified as low-risk PCa based on the NCCN classification showed a metabolic profile typical of Metabotype IV but with lower level of GlycA and GlycB. This patient died only 50 days after sample collection due to pancreatic cancer. On the other hand, patients classified as very high aggressiveness but that do not belong to Metabotype IV showed a survival time longer than 3 years and a lower Gleason score compared with patients belong to Metabotype IV. We were unable to record the date of death for 4 out of 7 patients belong to Metabotype IV. These patients were lost to follow-up at the hospital cancer center where they were recruited nor did they have any type of diagnostic test at a South African clinic or hospital. Considering the severe condition of these patients and the absence of registered diagnostic tests following the last visit, we assume the latter as a rough estimation of the time of survival. Almost all patients of Metabotype IV seem to have died within 1 year after the sample collection (Table 3).

**Table 3:** Demographics and clinical features of patients belonging to Metabotype IV and patients classified as very high aggressiveness that do not belong to Metabotype IV

Patient ID	Ethnicity	Collection date	Date of last visit	Date of death	Follow-up (day)^**c**^	Age (year)	PSA (ng/mL)	Gleason score	DRE	NCCN classification	GlycB rank^**d**^	Metabotype	Note
SAPC0159	Black	2017/03/01	2017/03/23		22	75.2	>5000	5+5	T3/T4	Metastatic	3	IV	
SAPC0090	Colored	2015/05/08		2015/06/27	50	78.2	6.3	3+3	T1c	Low	8	IV	a
SAPC0192	Colored	2017/07/31		2017/12/19	141	80.9	41.68	4+5	T2a	Very high	7	III	b
SAPC0080	Black	2014/10/31	2015/04/21		172	77.8	738	3+5	T4	Very high	2	IV	
SAPC0180	Black	2017/06/23	2017/12/12		172	58.1	>5000	5+5	T3/T4	Very high	1	IV	
SAPC0249	Colored	2018/03/25	2019/01/07		288	63	1070	5+4	T3	Very high	4	IV	
SAPC0078	White	2014/11/07		2015/10/31	358	74.3	135.79	5+5	T4	Very high	5	IV	
SAPC0193	Colored	2017/08/11	2019/04/26		623	63.5	48.85	3+4	T3	Very high	9	III	
SAPC0070	Colored	2014/01/08		2015/12/11	702	65.4	34.8	4+5	T2b	Very high	13	II	
SAPC0191	White	2017/07/28		2019/08/03	736	61.8	576	5+4	T3	Very high	6	IV	
SAPC0195	Black	2017/08/13	2020/01/30		900	87.2	26.53	4+5	T3	Very high	11	III	
SAPC0120	Black	2016/10/03	2019/09/30		1092	82.3	289.9	4+5	T3	Very high	14	II	
SAPC0108	Colored	2016/06/27		2020/04/17	1390	56.6	96.3	3+4	T4	Very high	12	III	
SAPC0076	Colored	2014/09/26	2020/06/04		2078	63.5	140.42	3+4	T3/T4	Very high	10	III	

^a^The patient was diagnosed with pancreas cancer 19 days after sample collection

^b^The patient died for bone metastasis 5 months after sample collection

^c^Number of days between the date of collection and the date of last visit at hospital or the date of death (if recorded)

^d^This number represent the rank of order of the GlycB values starting from the highest value

Noteworthy, we reported a few clues of possible differences of the prostate tissue inflammation among the metabotypes. Of 14 patients in Metabotype III, 3 patients had mild chronic inflammation and 2 patients had chronic inflammation reported on the histological exam of their prostate tissue. Of the 10 patients of Metabotype II, 2 patients had mild chronic inflammation, 3 patients had acute-on-chronic inflammation, and 1 had acute prostatitis on the histological exam.

### Metabolic profiling of PCa metabotypes

**Fig. 3:**
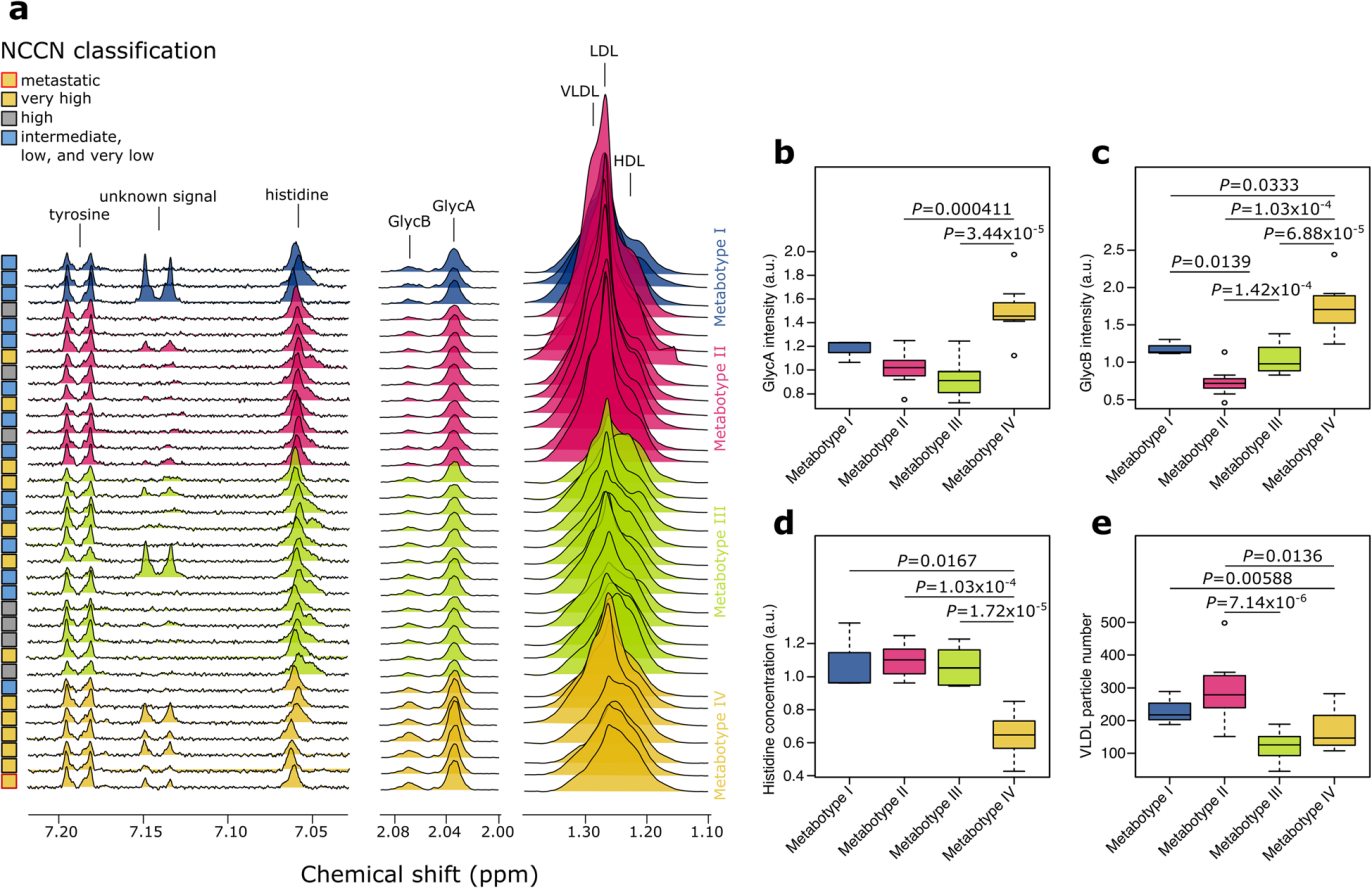
**a** NMR profiles of the plasma of three different spectral regions and Box-whiskers plots of the intensity of **b** GlycA and **c** GlycB, the concentration of **d** histidine and the number of **e** VLDL particles across the four metabotypes

The metabolic differences discriminating between among the four metabotypes appear to be clear (Fig. 2c). An overview of some discriminative NMR signals among the four metabotypes are showed in Fig. 3. Although we were aware of the low number of patients in this cohort, we built a supervised PLS model to evaluate the accuracy of the identification of the most aggressive metabotype (i.e., Metabotype IV) using the metabolic profile. Using a double cross-validation approach, we calculated an accuracy value of 91.2%, with a 95% coefficient interval of 86.0–94.1%. Next, we used the Wilcoxon rank-sum test to characterize this metabotype compared to the others (Supplementary Table S1). As previously mentioned, Metabotype IV is characterized by higher values of the inflammatory markers GlycA (*P* = 7.06 × 10^-6^; FDR = 7.24 × 10^−5^) and GlycB (*P* = 2.60 × 10^−6^; FDR = 3.56 × 10^−5^) and lower protein level (*P* = 9.15 × 10^−5^; FDR = 5.36 × 10^−4^). In addition, we detected a higher level of mannose (*P* = 4.45 × 10^−3^; FDR = 1.40 × 10^−2^), an important constituent of N-glycans of glycoproteins [38]. Mannose residues in N-glycans can be derived from either glycogen/glucose or mannose in the blood [38]. Moreover, we detected a reduced level of amino acids and their derivates in Metabotype IV. Among them, the reduction of histidine is the most significant (*P* = 3.72 × 10^−7^; FDR = 1.52 × 10^−5^). We also reported an unidentified metabolite at 7.14 ppm, with a hypothesized chemical similarity with tyrosine, prevalent in patients with very high aggressive PCa.

Similar to the previous analysis, we also investigated the rearrangement of the lipoprotein profile using the data from the application of the Bruker’s B.I.-LISA protocols (Supplementary Table S2). The Bruker’s B.I.-LISA protocols were used to characterize 114 parameters related to the lipoproteins, such as HDL, LDL, and VLDL, and their relative subclasses. We observed an association between Metabotype IV and lower level of apolipoprotein Apo-A1 (*P* = 2.67 × 10^−3^; FDR = 6.10 × 10^−2^) and Apo-A2 (*P* = 4.04 × 10^−3^; FDR = 5.32 × 10^−2^), attributable to a reduced HDL particle number (*P* = 1.28 × 10^−2^; FDR = 2.68 × 10^−1^), and higher level of triglycerides in LDL of smaller size, including LDL-1 (*P* = 4.45 × 10^−3^; FDR = 8.45 × 10^−2^) and LDL-2 (*P* = 1.40 × 10^−3^; FDR = 5.32 × 10^−2^). This finding completes the snapshot of Metabotype IV as a large regulator of the blood constituents, including metabolites, proteins, and lipoproteins with several implications for the role of inflammation as a confounding factor of PCa.

**Fig. 4:**
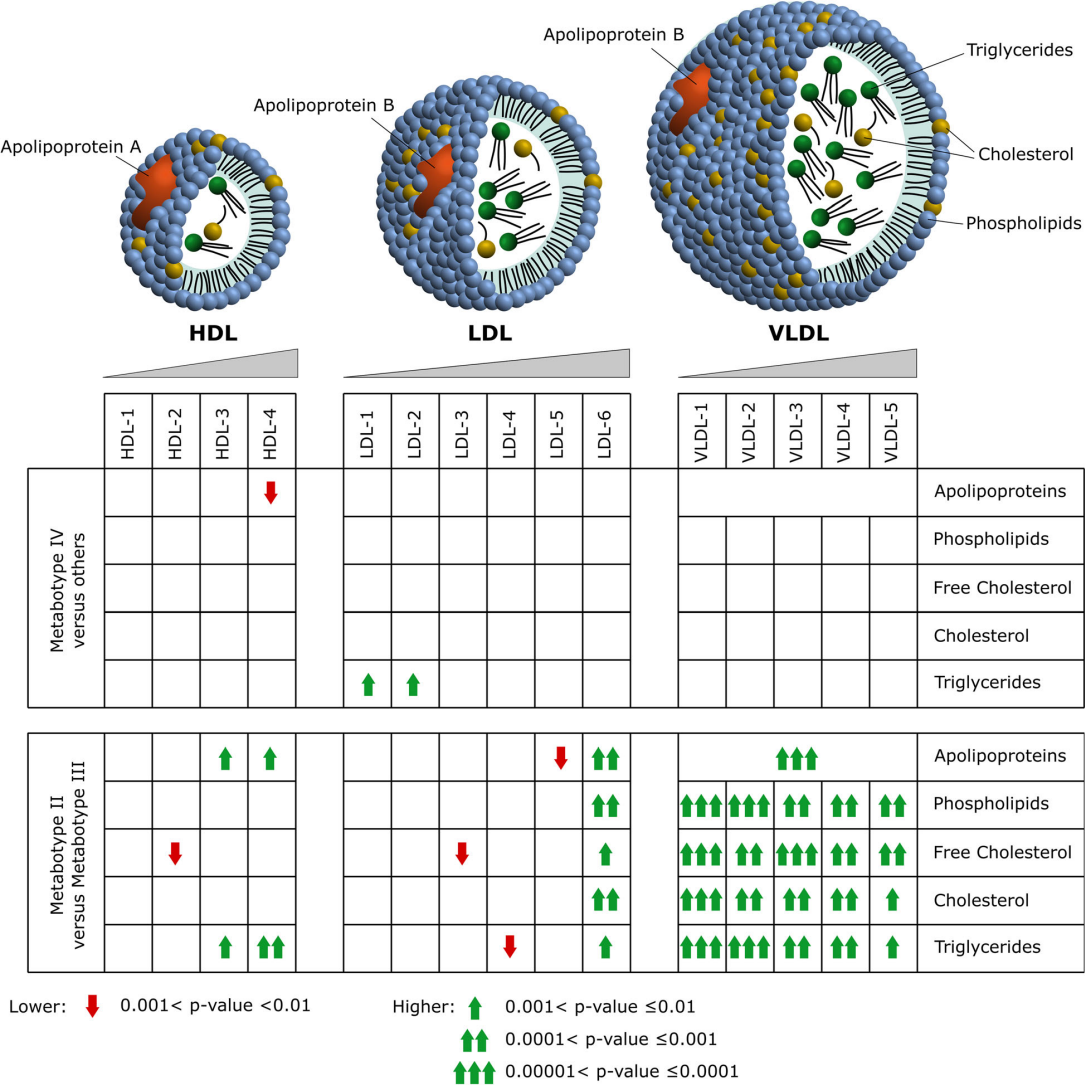
Graphics illustration of the changes of the lipoprotein profile

The levels of inflammatory NMR markers GlycA and GlycB have been shown to be highly correlated, as previously reported in the literature [36]. In our study, we reported a Spearman’s rank correlation rho of 0.59 (*P* = 7.16 × 10^−5^). However, the biological meaning of the differences between GlycA and GlycB has not yet been fully explored. In our cohort, we observed that two distinct metabotypes, Metabotype II and Metabotype III, had a similar level of GlycA but different levels of GlycB (*P* = 1.42 × 10^−4^; FDR = 6.46 × 10^−4^), with the latter showing the higher level (Supplementary Table S3). Moreover, Metabotype III seems to be associated with extremely reduced levels of lipids (Fig. 3a, e). We discovered a deep difference in the lipoprotein profile between Metabotype III and Metabotype II using the B.I.-LISA protocols (Supplementary Table S4). Figure 4 shows a graphical representation of the lipoprotein profile changes among metabotypes. Besides the evident reduction of VLDL, we also noted the lower values of Apo-A1 and Apo-A2 that could help to characterize the differences between GlycA and GlycB.

## Discussion

In this first metabolomic study of PCa conducted on an African population for metabolic biomarkers of inflammation, we observed in men with very high aggressive PCa higher levels of the NMR inflammatory markers GlycA and GlycB, indicating an increased concentration of positive APPs and/or a higher complexity of their glycan structures. Moreover, we noted a simultaneous reduction of the signal from protein source likely attributable to a reduction of the albumin level, a negative APP.

Interestingly, in our cohort, we discovered four distinct metabotypes associated with the aggressiveness of PCa, each one characterized by a unique metabolic fingerprint. A metabotype identified as a subgroup of patients with very high aggressive PCa (that we named Metabotype IV) was characterized by the highest values of GlycA and GlycB and by deep changes of the plasma metabolome. We observed a lower level of histidine that could reinforce our hypothesis of inflammatory processes underlying Metabotype IV. Indeed, histidine has been already associated with inflammatory processes and in particular, it has been negatively correlated with other inflammatory markers, such as IL-6 and CRP [39]. In addition, it has been reported that inflammation may alter the lipoprotein profile as well, for example modulating the HDL functions [40–42] as we observed. Recently, it has been shown that high levels of triglycerides and glucose and low levels of HDL cholesterol and Apo-A1 are related to increased PCa risk and its severity [43]. Moreover, low HDL was reported to be a risk and prognostic factor for PCa in several epidemiologic studies [44]. Elevated serum triglycerides were associated with an increased risk of PCa recurrence [45]. Lower levels of Apo-A1 and Apo-A2, and a higher level of triglycerides in LDL, reported in this study, are consistent with these processes and we suggest that inflammation could be a driving factor of the lipoprotein profile changes observed in Metabotype IV.

Although in vitro studies reported that aerobic glycolysis can be directly induced by an inflammatory microenvironment [46, 47], we did not observe any metabolic signature of the Warburg effect in the plasma of the patients with an inflammatory status associated with the PCa. Indeed, plasma metabolome do not necessarily reflect the Warburg effect present in the tumor tissue. As reported in a study on metastatic colorectal cancer (CRC) [4] where the Warburg effect is well-established, patients with mCRC showed lower serum levels of lactate and non-statistically significant changes of glucose compared to the controls. Liver gluconeogenesis [48] and insulin resistance associated with inflammation [49] may mask anaerobic dissimilation of glucose and lactate production.

Furthermore, we highlighted two other distinct metabotypes (i.e., Metabotype II and Metabotype III) characterized by large differences in the lipoprotein profile. Although anthropometric measures (e.g., body mass index) were not available in this study, patients with a reduced numbers of small LDL particles and higher concentrations of large LDL particles could be associated to an obese phenotype [50]. This fingerprint has been revealed in Metabotype II when compared to Metabotype III suggesting that patients in Metabotype II are characterized by higher body fat. Obesity causes systemic inflammation [51] and recently has been associated with higher level of GlycA [52, 53]. However, we noted a higher concentration of GlycB in Metabotype III, which is characterized by a higher number of patients with very high aggressive PCa compared to Metabotype II. No differences in the concentration of GlycA and higher levels of GlycB could be due to an elevated sialylation post-translational modification on glycosylated proteins. Complex biantennary glycoforms with α2,3-sialic acid have been associated with aggressive PCa [54–56]. Here, for the first time, we reported the association of lower level of the inflammatory NMR biomarker GlycB with a higher concentration of VLDL. Since the dietary intake can modulate the VLDL [50], this finding will further enrich the long-standing debate over the role of dietary fat in promoting PCa [57, 58].

In men diagnosed with PCa, the selection of the treatment, including the type of therapy and its aggressiveness, is often based on patient age and life expectancy. In an era of precision medicine, an estimate of the threat of disease and the benefit and the costs of intervention within the context of the patient’s characteristics and desires should be taken into consideration regarding the decision of the treatment. In this study, we identified a set of patients with very high aggressive PCa with extremely reduced survival time. These patients, belonging to Metabotype IV, are characterized by a similar metabolic profile predictable with high accuracy. Our results postulate that this subgroup may be most likely to benefit from combination therapy that associates the androgen deprivation in conjunction with drugs aiming to reduce the level of systemic inflammation. The life expectancy difference highlights the need to consider an appropriate medical treatment for patients within Metabotype IV. We hypothesize that these patients could largely benefit from daily treatment with corticosteroids to reduce the systemic inflammation improving the overall survival, along with the need for subsequent therapy. However, the only clinical settings of corticosteroids treatment with proved clinical utility in PCa treatment is in combination with abiraterone. We consider the lack of clinical investigation for almost all patients of the presence of distant metastasis as a limitation of this study, and we are aware that PCa could have spread to distant organs in the patients with very high-risk PCa belonging to Metabotype IV.

Some study limitations warrant mention. Our study is limited by the relatively small sample size and the lack of an independent cohort to validate the results which will need confirmation; yet, it provides the first metabolomic analysis of South African men with aggressive PCa. We also acknowledge that only the cohort from South Africa was evaluated. Therefore, we cannot generalize our results and draw any conclusions about ethnical and racial similarities or differences. Our results will require confirmation in larger, independent cohorts as well as across other ethnicities and races.

## Conclusions

This study has identified metabolic markers of inflammation and distinct metabotypes linked to aggressive PCa and aids in the urgent need for new precision medicine approaches aiming to profile aggressive and lethal PCa in African patients. For the first time, a portrait of the metabolic changes in African men with PCa has been delineated indicating a strong association between an inflammatory metabolic profile and aggressive forms of PCa.

Our findings indicate how the metabolic profile could be used to identify those patients with high level of inflammation, characterized by aggressive PCa and short life expectancy. Integrating a metabolomic analysis as a tool for patient stratification could be important for opening the door to the development of new therapy in African patients and in those patients with aggressive and lethal PCa, as they may benefit from therapeutic interventions, targeting the lowering of systemic inflammation. Whether similar metabotypes are present in other ethnicities will need further studies. Thus, further studies are necessary to better characterize this group of patients and determine the costs and benefits of corticosteroid treatment in terms of survival time and quality of life. The current project is still ongoing, and we are recruiting more patients with PCa to further validate our results in a larger, independent cohort.

## Supplementary Information

**Additional file 1: Table S1.** Statistical comparison between the metabolic profiles of Metabotype IV versus the others. **Table S2.** Statistical comparison between the lipoprotein profiles of Metabotype IV versus the others. **Table S3.** Statistical comparison between the metabolic profiles of Metabotype II versus Metabotype III. **Table S4.** Statistical comparison between the lipoprotein profiles of Metabotype II versus Metabotype III.

## Data Availability

The data that support the findings of this study are available on request from the corresponding author. The data are not publicly available due to privacy or ethical restrictions.

## Abbreviations

AA: African American
ADT: Androgen-deprivation therapy
APP: Acute-phase protein
BO: Bilateral orchidectomy
CRP: C-reactive protein
CRPC: Castration-resistant prostate cancer
CSPC: Castration-sensitive prostate cancer
EA: European American
FDR: False discovery rate
HDL: High-density lipoprotein
IDL: Intermediate-density lipoprotein
IL: Interleukin
IFN: Type I interferon
LDL: Low-density lipoprotein
Metabotype: Metabolic phenotype
mCRPC: Metastatic castration-resistant prostate cancer
NCCN: National Comprehensive Cancer Network
NMR: Nuclear magnetic resonance
NOESY: Nuclear Overhauser Effect SpectroscopY
PCa: Prostate cancer
PLS: Partial least-squares
PSA: Prostate-specific antigen
Q2: Predictive ability parameter
R2: Fit parameter
rho: Correlation coefficient
VLDL: Very low-density lipoprotein
