# Supplementary material for: Inflammatory metabolic profile of South African patients with prostate cancer
Source: Cancer Metab. 2021 Aug 3;9:29. doi: 10.1186/s40170-021-00265-6 (PMC8336341; doi:10.1186/s40170-021-00265-6)
Supplement: Supplementary file 1 — Additional file 1: Table S1. Statistical comparison between the metabolic profiles of Metabotype IV versus the others. Table S2. Statistical comparison between the lipoprotein profiles of Metabotype IV versus the others. Table S3. Statistical comparison between the metabolic profiles of Metabotype II versus Metabotype III. Table S4. Statistical comparison between the lipoprotein profiles of Metabotype II versus Metabotype III. [file 40170_2021_265_MOESM1_ESM.docx]

**Table S1.** Statistical comparison between the metabolic profiles of Metabotype IV *versus* the others.

| **Feature** | **Metabotype IV, median [IQR]** | **Others, median [IQR]** | **log change** | **p-value** | **FDR** |
| --- | --- | --- | --- | --- | --- |
| Histidine | 0.647 [0.566 0.732] | 1.067 [0.981 1.163] | -0.74 | 3.72x10^-7^ | 1.52x10^-5^ |
| Lipid (beta-CH2) | 0.743 [0.6 0.785] | 1.12 [0.989 1.239] | -0.69 | 7.44x10^-7^ | 1.52x10^-5^ |
| GlycB | 1.706 [1.524 1.89] | 0.959 [0.782 1.126] | 0.89 | 2.60x10^-6^ | 3.56x10^-5^ |
| GlycA | 1.455 [1.425 1.568] | 0.981 [0.892 1.075] | 0.61 | 7.06x10^-6^ | 7.24x10^-5^ |
| Valine | 0.62 [0.528 0.668] | 1.002 [0.915 1.244] | -0.77 | 6.73x10^-5^ | 4.60x10^-4^ |
| Leucine | 0.624 [0.487 0.764] | 1.065 [0.878 1.282] | -0.81 | 6.73x10^-5^ | 4.60x10^-4^ |
| Protein | 0.9 [0.87 0.939] | 1.021 [0.979 1.071] | -0.19 | 9.15x10^-5^ | 5.36x10^-4^ |
| Alanine | 0.706 [0.578 0.774] | 1.075 [0.89 1.234] | -0.69 | 2.10x10^-4^ | 1.07x10^-3^ |
| Isoleucine | 0.616 [0.548 0.811] | 1.076 [0.896 1.224] | -0.78 | 3.45x10^-4^ | 1.57x10^-3^ |
| Glutamine | 0.842 [0.747 0.976] | 1.098 [1.007 1.203] | -0.4 | 1.54x10^-3^ | 6.33x10^-3^ |
| Lipid (-CH3-) | 0.868 [0.826 0.946] | 1.062 [0.948 1.189] | -0.28 | 2.67x10^-3^ | 9.97x10^-3^ |
| Mannose | 1.368 [1.212 1.497] | 1.011 [0.754 1.173] | 0.46 | 4.45x10^-3^ | 1.40x10^-2^ |
| Threonine | 0.663 [0.54 0.829] | 1.042 [0.783 1.49] | -0.78 | 4.45x10^-3^ | 1.40x10^-2^ |
| 2-Hydroxyvalerate | 0.867 [0.853 0.938] | 1.053 [0.961 1.222] | -0.3 | 5.22x10^-3^ | 1.53x10^-2^ |
| 3-Hydroxybutyrate | 0.875 [0.812 0.899] | 1.029 [0.965 1.196] | -0.42 | 6.11x10^-3^ | 1.67x10^-2^ |
| Unsaturated lipid (-CH=CH-) | 0.769 [0.723 0.922] | 1.05 [0.904 1.332] | -0.46 | 8.27x10^-3^ | 1.99x10^-2^ |
| Glycorol phospholipid | 0.475 [0.276 0.577] | 1.24 [0.552 1.922] | -1.39 | 8.27x10^-3^ | 1.99x10^-2^ |
| Creatine | 0.493 [0.362 0.765] | 1.103 [0.637 1.575] | -1.08 | 1.10x10^-2^ | 2.51x10^-2^ |
| Pyroglutamate | 0.879 [0.864 0.951] | 1.023 [0.96 1.239] | -0.25 | 1.45x10^-2^ | 2.98x10^-2^ |
| Phospholipid | 1.184 [1.1 1.311] | 1.014 [0.926 1.118] | 0.23 | 1.45x10^-2^ | 2.98x10^-2^ |
| Unknown signal at 7.14 ppm | 13.361 [6.036 19.946] | 1 [1 1] | 0.79 | 2.11x10^-2^ | 4.12x10^-2^ |
| Isobutyrate | 0.643 [0.588 0.868] | 0.959 [0.848 1.202] | -0.35 | 3.89x10^-2^ | 7.25x10^-2^ |
| Glutamate | 0.702 [0.467 0.938] | 1.146 [0.664 1.378] | -0.87 | 4.85x10^-2^ | 8.64x10^-2^ |
| Pyruvate | 1.781 [1.131 2.386] | 1 [0.643 1.507] | 0.74 | 8.87x10^-2^ | 1.52x10^-1^ |
| Lipid (-(-CH2-)n-) | 0.825 [0.773 0.992] | 1.051 [0.892 1.512] | -0.43 | 9.74x10^-2^ | 1.60x10^-1^ |
| Lipid (alpha-CH2) | 0.518 [0.491 0.974] | 0.943 [0.671 1.98] | -0.86 | 1.17x10^-1^ | 1.84x10^-1^ |
| Methanol | 0.919 [0.682 1.255] | 1.244 [0.924 1.534] | -0.32 | 1.39x10^-1^ | 2.10x10^-1^ |
| Acetoacetate | 0.833 [0.643 0.94] | 0.958 [0.734 1.286] | -0.52 | 1.77x10^-1^ | 2.59x10^-1^ |
| Formate | 1.119 [0.951 1.352] | 1.023 [0.767 1.158] | 0.24 | 2.57x10^-1^ | 3.63x10^-1^ |
| 2-Hydroxybutyrate | 0.989 [0.854 1.332] | 0.933 [0.759 1.196] | 0.21 | 2.75x10^-1^ | 3.76x10^-1^ |
| 3-Hydroxyisovalerate | 0.484 [0.392 2.729] | 0.663 [0.489 3.622] | -0.03 | 3.35x10^-1^ | 4.29x10^-1^ |
| Lipid (=CH-CH2-CH=) | 0.95 [0.819 1.004] | 1.05 [0.86 1.102] | -0.1 | 3.35x10^-1^ | 4.29x10^-1^ |
| Glycine | 1.003 [0.886 1.145] | 1.109 [0.979 1.193] | -0.14 | 4.27x10^-1^ | 5.30x10^-1^ |
| Cholesterol backbone | 1.047 [0.912 1.146] | 1.131 [0.93 1.324] | -0.11 | 5.04x10^-1^ | 6.08x10^-1^ |
| Glucose | 1.015 [0.865 1.093] | 1 [0.898 1.247] | -0.13 | 5.59x10^-1^ | 6.51x10^-1^ |
| Lactate | 1.388 [0.738 1.634] | 0.869 [0.698 1.334] | 0.27 | 5.88x10^-1^ | 6.51x10^-1^ |
| Citrate | 0.969 [0.8 1.195] | 1.042 [0.805 1.285] | -0.1 | 5.88x10^-1^ | 6.51x10^-1^ |
| Creatinine | 0.974 [0.873 1.149] | 1.004 [0.807 1.14] | -0.03 | 7.39x10^-1^ | 7.97x10^-1^ |
| Phenylalanine | 0.958 [0.821 1.113] | 0.95 [0.799 1.111] | 0.03 | 8.03x10^-1^ | 8.44x10^-1^ |
| Acetate | 1.053 [0.873 1.247] | 1.004 [0.902 1.255] | -0.05 | 9.01x10^-1^ | 9.23x10^-1^ |
| Tyrosine | 1.077 [0.87 1.186] | 1.043 [0.865 1.148] | 0.01 | 1.00 | 1.00 |

**Table S2.** Statistical comparison between the lipoprotein profiles of Metabotype IV *versus* the others.

| **Feature** | **Metabotype IV, median [IQR]** | **Others, median [IQR]** | **log change** | **p-value** | **FDR** |
| --- | --- | --- | --- | --- | --- |
| Main_Parameters_Apo.A2 | 25.01 [22.865 26.685] | 30.93 [27.97 33.845] | -0.32 | 1.04x10^-3^ | 5.32x10^-2^ |
| HDL_Subfractions_Apo.A2_HDL.4 | 12.15 [11.055 13.345] | 18.24 [14.905 21.8] | -0.6 | 1.04x10^-3^ | 5.32x10^-2^ |
| LDL_Subfractions_Triglycerides_LDL.2 | 3.59 [3.33 3.905] | 2.08 [1.825 2.755] | 0.63 | 1.40x10^-3^ | 5.32x10^-2^ |
| MainFractions_Apo.A2_HDL | 26.37 [24.41 28.245] | 31.88 [29.105 34.71] | -0.31 | 1.87x10^-3^ | 5.34x10^-2^ |
| HDL_Subfractions_Apo.A1_HDL.4 | 54.24 [48.105 57.045] | 64.89 [60.085 76.18] | -0.35 | 2.67x10^-3^ | 6.10x10^-2^ |
| LDL_Subfractions_Triglycerides_LDL.1 | 8.15 [7.56 9.31] | 5.15 [3.7 7.2] | 0.57 | 4.45x10^-3^ | 8.45x10^-2^ |
| HDL_Subfractions_Phospholipid_HDL.4 | 18.97 [18.37 19.995] | 22.74 [20.58 27.65] | -0.33 | 5.22x10^-3^ | 8.50x10^-2^ |
| MainFractions_Triglycerides_LDL | 25.6 [23.305 28.935] | 20.07 [16.775 23.18] | 0.38 | 8.27x10^-3^ | 1.18x10^-1^ |
| MainFractions_Phospholipids_IDL | 2.88 [2.1 5.535] | 7.1 [4.77 11.555] | -1.13 | 9.37x10^-3^ | 1.19x10^-1^ |
| HDL_Subfractions_Cholesterol_HDL.4 | 13.87 [11.825 14.56] | 18.07 [14.09 22.105] | -0.44 | 1.06x10^-2^ | 1.21x10^-1^ |
| VLDL_Subfractions_Cholesterol_VLDL.1 | 2.71 [1.69 4.625] | 7.76 [4.5 17.16] | -1.52 | 1.43x10^-2^ | 1.48x10^-1^ |
| Main_Parameters_Cholesterol | 151.07 [139.25 184.675] | 209.08 [183.615 223.545] | -0.32 | 1.89x10^-2^ | 1.78x10^-1^ |
| VLDL_Subfractions_FreeCholesterol_VLDL.1 | 0.5 [0.355 1.135] | 2.84 [1.345 6.68] | -2.05 | 2.02x10^-2^ | 1.78x10^-1^ |
| LDL.5_Particle_Number | 122.22 [44.215 173.705] | 235.58 [150.9 303.1] | -0.82 | 2.75x10^-2^ | 2.08x10^-1^ |
| LDL_Subfractions_Apo.B_LDL.5 | 6.72 [2.43 9.555] | 12.96 [8.3 16.67] | -0.82 | 2.75x10^-2^ | 2.08x10^-1^ |
| VLDL_Subfractions_Phospholipids_VLDL.1 | 2.89 [2.05 3.805] | 7.51 [3.32 14.185] | -1.4 | 3.09x10^-2^ | 2.08x10^-1^ |
| VLDL_Subfractions_Cholesterol_VLDL.2 | 2.38 [1.82 2.735] | 3.91 [2.935 5.69] | -0.73 | 3.32x10^-2^ | 2.08x10^-1^ |
| LDL_Subfractions_Cholesterol_LDL.5 | 7.27 [1.465 11.7] | 18.74 [10.945 22.83] | -1.01 | 3.32x10^-2^ | 2.08x10^-1^ |
| LDL_Subfractions_Phospholipids_LDL.5 | 4.84 [1.515 6.935] | 9.85 [6.405 11.875] | -0.87 | 3.47x10^-2^ | 2.08x10^-1^ |
| VLDL_Subfractions_Triglycerides_VLDL.1 | 11.87 [9.42 26.39] | 39.89 [19.25 92.63] | -1.53 | 4.35x10^-2^ | 2.36x10^-1^ |
| HDL_Subfractions_Triglycerides_HDL.1 | 4.34 [3.785 5.4] | 3.33 [2.37 3.97] | 0.4 | 4.35x10^-2^ | 2.36x10^-1^ |
| MainFractions_Apo.A1_HDL | 126.51 [118.6 131.56] | 138.97 [133.715 147.975] | -0.14 | 4.85x10^-2^ | 2.51x10^-1^ |
| Main_Parameters_Apo.A1 | 128.49 [120.11 132.985] | 137.99 [131.55 147.33] | -0.13 | 5.98x10^-2^ | 2.97x10^-1^ |
| LDL_Subfractions_FreeCholesterol_LDL.5 | 2.63 [1.05 3.635] | 4.45 [2.98 5.795] | -0.66 | 7.36x10^-2^ | 3.50x10^-1^ |
| HDL_Subfractions_Triglycerides_HDL.2 | 2.14 [1.96 2.345] | 1.7 [1.365 2.22] | 0.32 | 8.07x10^-2^ | 3.68x10^-1^ |
| LDL_Subfractions_Triglycerides_LDL.3 | 2.9 [2.51 3.37] | 2.4 [1.705 2.765] | 0.36 | 8.45x10^-2^ | 3.68x10^-1^ |
| LDL_Subfractions_Cholesterol_LDL.4 | 1.51 [0 4.95] | 8.94 [2.735 13.525] | -1.03 | 8.71x10^-2^ | 3.68x10^-1^ |
| MainFractions_Triglycerides_IDL | 4.43 [2.895 7.345] | 9.89 [5.215 25.96] | -1.39 | 9.25x10^-2^ | 3.70x10^-1^ |
| Main_Parameters_LDL.Chol | 70.08 [51.52 95.205] | 104.2 [82.98 116.18] | -0.38 | 9.74x10^-2^ | 3.70x10^-1^ |
| MainFractions_Cholesterol_LDL | 70.08 [51.52 95.205] | 104.2 [82.98 116.18] | -0.38 | 9.74x10^-2^ | 3.70x10^-1^ |
| LDL_Subfractions_Triglycerides_LDL.4 | 2.58 [2.16 2.75] | 1.55 [1.11 2.14] | 0.56 | 1.06x10^-1^ | 3.88x10^-1^ |
| MainFractions_Phospholipids_VLDL | 14.07 [11.885 23.41] | 23.72 [16.435 39.41] | -0.56 | 1.17x10^-1^ | 3.95x10^-1^ |
| LDL.4_Particle_Number | 34.66 [0 72.185] | 119.2 [41.125 153.81] | -0.85 | 1.24x10^-1^ | 3.95x10^-1^ |
| LDL_Subfractions_Phospholipids_LDL.4 | 2.14 [0 3.865] | 5.41 [1.77 7.75] | -0.82 | 1.24x10^-1^ | 3.95x10^-1^ |
| LDL_Subfractions_Apo.B_LDL.4 | 1.91 [0 3.97] | 6.56 [2.265 8.46] | -0.85 | 1.24x10^-1^ | 3.95x10^-1^ |
| MainFractions_FreeCholesterol_VLDL | 6.27 [5.155 10.92] | 11.05 [7.74 17.9] | -0.58 | 1.25x10^-1^ | 3.95x10^-1^ |
| LDL.Chol_HDL.Chol | 1.48 [0.985 1.8] | 1.82 [1.61 2.12] | -0.23 | 1.47x10^-1^ | 3.95x10^-1^ |
| MainFractions_Cholesterol_VLDL | 13.65 [11.82 24.19] | 26.46 [17.545 40.495] | -0.57 | 1.51x10^-1^ | 3.95x10^-1^ |
| LDL_Subfractions_Phospholipids_LDL.6 | 12.38 [9.875 17.385] | 16.01 [13.97 19.735] | -0.31 | 1.51x10^-1^ | 3.95x10^-1^ |
| HDL_Subfractions_Phospholipid_HDL.1 | 25.88 [23.355 28.415] | 20.69 [14.64 27.49] | 0.29 | 1.51x10^-1^ | 3.95x10^-1^ |
| MainFractions_FreeCholesterol_LDL | 22.25 [19.255 27.525] | 28.41 [24.245 32.88] | -0.21 | 1.60x10^-1^ | 3.95x10^-1^ |
| Main_Parameters_Apo.B100 | 69.62 [62.005 86.355] | 91.46 [76.055 99.625] | -0.21 | 1.63x10^-1^ | 3.95x10^-1^ |
| Total_Particle_Number | 1265.89 [1127.435 1570.115] | 1663.05 [1382.915 1811.48] | -0.21 | 1.63x10^-1^ | 3.95x10^-1^ |
| MainFractions_Triglycerides_VLDL | 60.34 [40.14 72.935] | 74.42 [49.29 161.715] | -0.77 | 1.63x10^-1^ | 3.95x10^-1^ |
| LDL_Subfractions_Cholesterol_LDL.6 | 21.31 [16.14 31.8] | 29.34 [24.955 37.035] | -0.38 | 1.63x10^-1^ | 3.95x10^-1^ |
| LDL_Subfractions_Apo.B_LDL.1 | 13.34 [12.155 13.715] | 11.37 [8.335 13.33] | 0.18 | 1.73x10^-1^ | 3.95x10^-1^ |
| LDL_Particle_Number | 1006.8 [780.95 1357.535] | 1329.88 [1132.015 1424.785] | -0.23 | 1.77x10^-1^ | 3.95x10^-1^ |
| LDL.1_Particle_Number | 242.55 [220.995 249.37] | 206.72 [151.57 242.355] | 0.18 | 1.77x10^-1^ | 3.95x10^-1^ |
| MainFractions_Phospholipids_LDL | 46.19 [37.155 58.085] | 59.18 [47.095 66.04] | -0.2 | 1.77x10^-1^ | 3.95x10^-1^ |
| MainFractions_Apo.B_LDL | 55.37 [42.95 74.66] | 73.14 [62.255 78.36] | -0.23 | 1.77x10^-1^ | 3.95x10^-1^ |
| HDL_Subfractions_Cholesterol_HDL.1 | 22.1 [19.685 24.495] | 18.78 [13.855 23.415] | 0.24 | 1.77x10^-1^ | 3.95x10^-1^ |
| VLDL_Subfractions_FreeCholesterol_VLDL.2 | 1.17 [1.04 1.43] | 1.91 [1.16 2.945] | -0.58 | 1.87x10^-1^ | 4.09x10^-1^ |
| MainFractions_FreeCholesterol_IDL | 2.34 [1.975 4.385] | 4.03 [2.78 5.695] | -0.46 | 2.01x10^-1^ | 4.28x10^-1^ |
| LDL.6_Particle_Number | 325.77 [240.52 484.46] | 450.55 [346.56 564.44] | -0.39 | 2.06x10^-1^ | 4.28x10^-1^ |
| LDL_Subfractions_Apo.B_LDL.6 | 17.92 [13.23 26.645] | 24.78 [19.06 31.045] | -0.39 | 2.06x10^-1^ | 4.28x10^-1^ |
| VLDL_Subfractions_Phospholipids_VLDL.2 | 2.64 [2.47 3.195] | 3.8 [2.465 5.65] | -0.47 | 2.17x10^-1^ | 4.41x10^-1^ |
| Main_Parameters_Triglycerides | 96.35 [80.635 122.505] | 114.96 [91.02 219.825] | -0.57 | 2.39x10^-1^ | 4.78x10^-1^ |
| MainFractions_Triglycerides_HDL | 10.91 [9.755 11.665] | 8.54 [7.13 12.12] | 0.14 | 2.57x10^-1^ | 4.92x10^-1^ |
| LDL_Subfractions_FreeCholesterol_LDL.4 | 1.95 [0.545 2.795] | 2.95 [1.57 4.345] | -0.5 | 2.59x10^-1^ | 4.92x10^-1^ |
| HDL_Subfractions_Apo.A2_HDL.3 | 5.51 [5.165 5.945] | 6.01 [5.535 6.885] | -0.19 | 2.59x10^-1^ | 4.92x10^-1^ |
| MainFractions_Cholesterol_IDL | 9.21 [7.735 15.675] | 14.04 [9.71 18.84] | -0.35 | 2.75x10^-1^ | 5.14x10^-1^ |
| LDL_Subfractions_FreeCholesterol_LDL.1 | 6.71 [5.86 7.34] | 5.47 [4.495 7.21] | 0.11 | 2.87x10^-1^ | 5.28x10^-1^ |
| VLDL_Subfractions_FreeCholesterol_VLDL.5 | 0.39 [0.35 1.16] | 0.93 [0.575 1.565] | -0.59 | 2.94x10^-1^ | 5.32x10^-1^ |
| LDL_Subfractions_Phospholipids_LDL.1 | 13.28 [11.57 13.34] | 11.24 [8.925 13.96] | 0.1 | 3.07x10^-1^ | 5.46x10^-1^ |
| Main_Parameters_HDL.Chol | 52.7 [48.33 54.175] | 55.73 [51.2 62.19] | -0.1 | 3.35x10^-1^ | 5.79x10^-1^ |
| MainFractions_Cholesterol_HDL | 52.7 [48.33 54.175] | 55.73 [51.2 62.19] | -0.1 | 3.35x10^-1^ | 5.79x10^-1^ |
| LDL_Subfractions_FreeCholesterol_LDL.6 | 5.21 [3.14 7.33] | 6.48 [5.045 7.83] | -0.27 | 3.49x10^-1^ | 5.90x10^-1^ |
| LDL_Subfractions_FreeCholesterol_LDL.2 | 5.75 [5.19 6.645] | 5.35 [4.185 6.4] | 0.18 | 3.57x10^-1^ | 5.90x10^-1^ |
| HDL_Subfractions_Triglycerides_HDL.3 | 2.21 [2.09 2.45] | 1.71 [1.585 2.645] | 0.17 | 3.57x10^-1^ | 5.90x10^-1^ |
| HDL_Subfractions_FreeCholesterol_HDL.3 | 1.4 [1.325 1.605] | 1.67 [1.375 1.95] | -0.1 | 3.71x10^-1^ | 6.04x10^-1^ |
| VLDL_Subfractions_Triglycerides_VLDL.2 | 9.48 [9.145 12.295] | 12.3 [7.77 20.28] | -0.35 | 4.03x10^-1^ | 6.26x10^-1^ |
| VLDL_Subfractions_Triglycerides_VLDL.4 | 11.24 [8.685 16.995] | 10.15 [7.525 13.39] | 0.26 | 4.03x10^-1^ | 6.26x10^-1^ |
| HDL_Subfractions_Triglycerides_HDL.4 | 2.8 [2.31 3.11] | 2.86 [2.515 3.98] | -0.23 | 4.03x10^-1^ | 6.26x10^-1^ |
| VLDL_Subfractions_FreeCholesterol_VLDL.3 | 1.11 [1.075 2.275] | 2.12 [1.16 3.395] | -0.42 | 4.06x10^-1^ | 6.26x10^-1^ |
| Apo.B100_Apo.A1 | 0.57 [0.475 0.68] | 0.66 [0.56 0.745] | -0.06 | 4.18x10^-1^ | 6.35x10^-1^ |
| VLDL_Subfractions_Cholesterol_VLDL.3 | 2.67 [2.43 5.15] | 4.67 [2.66 6.615] | -0.32 | 4.56x10^-1^ | 6.84x10^-1^ |
| VLDL_Subfractions_Triglycerides_VLDL.5 | 3.31 [2.555 3.66] | 3.01 [2.105 3.655] | 0.19 | 4.69x10^-1^ | 6.94x10^-1^ |
| HDL_Subfractions_FreeCholesterol_HDL.2 | 1.75 [1.495 1.82] | 1.57 [1.03 1.81] | 0.1 | 5.23x10^-1^ | 7.55x10^-1^ |
| HDL_Subfractions_Apo.A2_HDL.1 | 2.93 [2.72 3.645] | 2.86 [2.115 3.64] | 0.12 | 5.23x10^-1^ | 7.55x10^-1^ |
| HDL_Subfractions_Cholesterol_HDL.3 | 8.81 [8.215 9.26] | 9 [8.4 10.165] | -0.05 | 5.31x10^-1^ | 7.55x10^-1^ |
| LDL_Subfractions_Triglycerides_LDL.6 | 5.11 [4.85 7.015] | 5.12 [4.355 6.655] | 0.11 | 5.37x10^-1^ | 7.55x10^-1^ |
| HDL_Subfractions_FreeCholesterol_HDL.4 | 2.4 [2.05 2.655] | 2.42 [1.69 3.47] | -0.22 | 5.59x10^-1^ | 7.76x10^-1^ |
| VLDL_Subfractions_Phospholipids_VLDL.3 | 3.31 [2.965 5.41] | 4.97 [3.11 6.755] | -0.22 | 5.65x10^-1^ | 7.76x10^-1^ |
| LDL.2_Particle_Number | 166.03 [151.33 208.055] | 164.37 [139.665 205.405] | 0.11 | 5.88x10^-1^ | 7.84x10^-1^ |
| LDL_Subfractions_Apo.B_LDL.2 | 9.13 [8.32 11.44] | 9.04 [7.68 11.295] | 0.11 | 5.88x10^-1^ | 7.84x10^-1^ |
| VLDL_Subfractions_FreeCholesterol_VLDL.4 | 1.52 [1.075 3.665] | 2.14 [1.405 3.315] | -0.14 | 6.09x10^-1^ | 7.84x10^-1^ |
| LDL_Subfractions_Triglycerides_LDL.5 | 2.56 [2.1 3.05] | 2.94 [2.205 3.695] | -0.07 | 6.09x10^-1^ | 7.84x10^-1^ |
| HDL_Subfractions_Apo.A1_HDL.1 | 27.97 [26.28 30.92] | 26.72 [18.345 36.18] | 0.15 | 6.17x10^-1^ | 7.84x10^-1^ |
| VLDL_Subfractions_Cholesterol_VLDL.4 | 4.74 [3.605 8.725] | 5.18 [4.165 7.74] | 0 | 6.39x10^-1^ | 7.84x10^-1^ |
| VLDL_Subfractions_Cholesterol_VLDL.5 | 1.21 [0.59 1.64] | 1.51 [0.84 1.88] | -0.06 | 6.39x10^-1^ | 7.84x10^-1^ |
| VLDL_Particle_Number | 146.61 [124.635 216.05] | 174.76 [125.635 260.425] | -0.19 | 6.47x10^-1^ | 7.84x10^-1^ |
| IDL_Particle_Number | 85.1 [82.795 107.41] | 93.81 [66.715 125.515] | -0.12 | 6.47x10^-1^ | 7.84x10^-1^ |
| MainFractions_Apo.B_VLDL | 8.06 [6.855 11.885] | 9.61 [6.91 14.32] | -0.19 | 6.47x10^-1^ | 7.84x10^-1^ |
| MainFractions_Apo.B_IDL | 4.68 [4.555 5.905] | 5.16 [3.67 6.9] | -0.12 | 6.47x10^-1^ | 7.84x10^-1^ |
| LDL_Subfractions_Cholesterol_LDL.3 | 9.6 [6.69 12.495] | 13.12 [7.465 15.125] | -0.13 | 6.70x10^-1^ | 8.04x10^-1^ |
| MainFractions_FreeCholesterol_HDL | 10.57 [10.04 12.075] | 9.66 [9.24 12.95] | 0.06 | 7.01x10^-1^ | 8.24x10^-1^ |
| HDL_Subfractions_FreeCholesterol_HDL.1 | 3.98 [3.115 4.53] | 3.75 [2.475 4.825] | 0.02 | 7.01x10^-1^ | 8.24x10^-1^ |
| LDL_Subfractions_Phospholipids_LDL.2 | 8.86 [8.125 10.94] | 8.8 [7.225 11.315] | 0.09 | 7.39x10^-1^ | 8.60x10^-1^ |
| LDL_Subfractions_Apo.B_LDL.3 | 6.96 [5.93 8.795] | 7.25 [5.035 9.525] | 0.11 | 7.66x10^-1^ | 8.79x10^-1^ |
| LDL.3_Particle_Number | 126.58 [107.8 159.865] | 131.79 [91.545 173.235] | 0.11 | 7.71x10^-1^ | 8.79x10^-1^ |
| LDL_Subfractions_FreeCholesterol_LDL.3 | 3.79 [3.595 4.735] | 4.21 [2.53 5.035] | 0.15 | 7.98x10^-1^ | 8.97x10^-1^ |
| LDL_Subfractions_Cholesterol_LDL.2 | 14.59 [13.305 19.375] | 16.89 [13.345 20.83] | -0.01 | 8.03x10^-1^ | 8.97x10^-1^ |
| HDL_Subfractions_Apo.A1_HDL.3 | 24.55 [22.595 25.55] | 23.88 [22.23 27.09] | -0.05 | 8.15x10^-1^ | 9.02x10^-1^ |
| HDL_Subfractions_Phospholipid_HDL.2 | 13.27 [12.895 14.535] | 14.26 [10.64 15.715] | 0.07 | 8.35x10^-1^ | 9.15x10^-1^ |
| VLDL_Subfractions_Phospholipids_VLDL.5 | 1.36 [1 2.415] | 1.97 [1.055 2.29] | -0.06 | 8.48x10^-1^ | 9.21x10^-1^ |
| HDL_Subfractions_Apo.A1_HDL.2 | 19.84 [15.235 20.365] | 17.65 [15.25 20.59] | 0.02 | 8.98x10^-1^ | 9.66x10^-1^ |
| VLDL_Subfractions_Phospholipids_VLDL.4 | 4.86 [3.69 7.7] | 4.87 [3.93 6.625] | 0.1 | 9.15x10^-1^ | 9.75x10^-1^ |
| LDL_Subfractions_Phospholipids_LDL.3 | 6.36 [5.4 7.79] | 7.63 [4.78 8.565] | 0.05 | 9.32x10^-1^ | 9.76x10^-1^ |
| HDL_Subfractions_Cholesterol_HDL.2 | 8.36 [7.58 8.71] | 8.56 [6.67 10.025] | -0.01 | 9.34x10^-1^ | 9.76x10^-1^ |
| VLDL_Subfractions_Triglycerides_VLDL.3 | 9.86 [9.56 16.12] | 11.94 [9.045 19.305] | -0.03 | 9.66x10^-1^ | 9.84x10^-1^ |
| MainFractions_Phospholipids_HDL | 73.02 [67.19 75.64] | 72.67 [65.82 80.31] | -0.01 | 9.67x10^-1^ | 9.84x10^-1^ |
| LDL_Subfractions_Cholesterol_LDL.1 | 20.18 [17.485 21.32] | 18.55 [14.815 24.82] | -0.07 | 9.67x10^-1^ | 9.84x10^-1^ |
| HDL_Subfractions_Phospholipid_HDL.3 | 14.39 [13.985 15.93] | 14.33 [13.63 16.59] | 0 | 9.83x10^-1^ | 9.92x10^-1^ |
| HDL_Subfractions_Apo.A2_HDL.2 | 3.83 [3.415 4.015] | 3.61 [3.125 4.435] | -0.06 | 1.00 | 1.00 |

**Table S3.** Statistical comparison between the metabolic profiles of Metabotype II *versus* Metabotype III.

| **Feature** | **Metabotype II, median [IQR]** | **Metabotype III, median [IQR]** | **log change** | **p-value** | **FDR** |
| --- | --- | --- | --- | --- | --- |
| Pyroglutamate | 1.28 [1.226 1.343] | 0.974 [0.943 1.011] | 0.45 | 1.02x10^-6^ | 1.05x10^-5^ |
| 2-Hydroxyvalerate | 1.263 [1.21 1.325] | 0.961 [0.93 0.997] | 0.45 | 1.02x10^-6^ | 1.05x10^-5^ |
| Unsaturated lipid (-CH=CH-) | 1.388 [1.331 1.469] | 0.917 [0.838 0.998] | 0.64 | 1.02x10^-6^ | 1.05x10^-5^ |
| Lipid (-(-CH2-)n-) | 1.569 [1.51 1.742] | 0.892 [0.776 1.003] | 0.91 | 1.02x10^-6^ | 1.05x10^-5^ |
| Lipid (-CH3-) | 1.226 [1.183 1.272] | 0.948 [0.938 1.034] | 0.33 | 2.04x10^-6^ | 1.67x10^-5^ |
| Lipid (alpha-CH2) | 2.144 [1.957 2.631] | 0.671 [0.494 0.863] | 1.76 | 7.14x10^-6^ | 4.18x10^-5^ |
| Glycorol phospholipid | 2.084 [1.919 2.643] | 0.552 [0.401 0.809] | 1.73 | 7.14x10^-6^ | 4.18x10^-5^ |
| Threonine | 1.596 [1.341 1.786] | 0.783 [0.741 0.96] | 0.96 | 4.59x10^-5^ | 2.35x10^-4^ |
| GlycB | 0.72 [0.661 0.783] | 0.978 [0.903 1.167] | -0.5 | 1.42x10^-4^ | 6.46x10^-4^ |
| Lipid (beta-CH2) | 1.288 [1.193 1.36] | 1.032 [0.9 1.121] | 0.36 | 1.98x10^-4^ | 8.11x10^-4^ |
| 3-Hydroxybutyrate | 1.28 [1.183 1.351] | 0.979 [0.949 1.017] | 0.4 | 3.73x10^-4^ | 1.39x10^-3^ |
| Lipid (=CH-CH2-CH=) | 1.111 [1.08 1.156] | 0.957 [0.818 1.046] | 0.28 | 1.49x10^-3^ | 5.10x10^-3^ |
| Cholesterol backbone | 1.324 [1.228 1.43] | 1.021 [0.851 1.149] | 0.37 | 1.91x10^-3^ | 6.03x10^-3^ |
| Methanol | 0.978 [0.826 1.148] | 1.41 [1.091 1.572] | -0.46 | 2.20x10^-2^ | 6.45x10^-2^ |
| Pyruvate | 1.362 [0.789 1.844] | 0.918 [0.581 1.09] | 0.75 | 4.17x10^-2^ | 1.14x10^-1^ |
| Phenylalanine | 1.003 [0.942 1.072] | 0.834 [0.669 1.01] | 0.28 | 4.84x10^-2^ | 1.24x10^-1^ |
| Isoleucine | 1.146 [1.062 1.254] | 0.911 [0.777 1.182] | 0.28 | 8.41x10^-2^ | 2.03x10^-1^ |
| GlycA | 1.019 [0.96 1.073] | 0.913 [0.827 0.979] | 0.13 | 9.56x10^-2^ | 2.18x10^-1^ |
| Creatine | 1.401 [0.958 1.613] | 0.931 [0.45 1.289] | 0.33 | 1.08x10^-1^ | 2.34x10^-1^ |
| Mannose | 1.11 [0.949 1.27] | 0.977 [0.714 1.076] | 0.25 | 1.22x10^-1^ | 2.51x10^-1^ |
| Valine | 1.01 [0.936 1.284] | 0.956 [0.806 1.069] | 0.19 | 1.38x10^-1^ | 2.68x10^-1^ |
| 3-Hydroxyisovalerate | 0.568 [0.479 0.635] | 1.799 [0.498 4.786] | -1.53 | 1.72x10^-1^ | 3.21x10^-1^ |
| Glucose | 1.011 [0.925 1.471] | 0.911 [0.889 1.124] | 0.28 | 1.92x10^-1^ | 3.41x10^-1^ |
| Glutamine | 1.157 [1.108 1.204] | 1.07 [0.999 1.213] | 0.07 | 2.35x10^-1^ | 4.01x10^-1^ |
| Acetate | 0.956 [0.895 1.125] | 1.149 [0.959 1.291] | -0.15 | 3.41x10^-1^ | 5.59x10^-1^ |
| Formate | 0.979 [0.758 1.077] | 1.051 [0.716 1.253] | -0.18 | 3.71x10^-1^ | 5.85x10^-1^ |
| Glutamate | 0.802 [0.46 1.302] | 1.175 [0.814 1.223] | -0.23 | 4.03x10^-1^ | 6.12x10^-1^ |
| Glycine | 1.083 [0.933 1.158] | 1.151 [0.99 1.195] | -0.1 | 4.37x10^-1^ | 6.39x10^-1^ |
| Histidine | 1.101 [1.029 1.16] | 1.053 [0.967 1.151] | 0.04 | 4.72x10^-1^ | 6.67x10^-1^ |
| Creatinine | 1.023 [0.906 1.071] | 0.922 [0.754 1.126] | 0.09 | 5.08x10^-1^ | 6.72x10^-1^ |
| Leucine | 1.069 [0.941 1.199] | 0.94 [0.842 1.145] | 0.1 | 5.08x10^-1^ | 6.72x10^-1^ |
| Tyrosine | 1.044 [0.928 1.109] | 0.918 [0.784 1.1] | 0.11 | 5.46x10^-1^ | 6.99x10^-1^ |
| Alanine | 1.019 [0.856 1.274] | 0.95 [0.879 1.126] | 0.16 | 5.85x10^-1^ | 7.27x10^-1^ |
| 2-Hydroxybutyrate | 1.024 [0.746 1.18] | 0.865 [0.806 1.063] | 0.04 | 7.96x10^-1^ | 9.31x10^-1^ |
| Protein | 1.013 [0.962 1.072] | 1.028 [0.993 1.064] | 0 | 7.96x10^-1^ | 9.31x10^-1^ |
| Unknown signal at 7.14 ppm | 1 [1 1] | 1 [1 1] | -1.32 | 8.37x10^-1^ | 9.31x10^-1^ |
| Citrate.left | 1.003 [0.778 1.279] | 1.035 [0.819 1.249] | -0.01 | 8.41x10^-1^ | 9.31x10^-1^ |
| Lactate | 0.79 [0.689 1.483] | 0.82 [0.709 1.035] | 0.21 | 8.86x10^-1^ | 9.31x10^-1^ |
| Acetoacetate | 0.943 [0.724 1.231] | 0.904 [0.659 1.244] | -0.18 | 8.86x10^-1^ | 9.31x10^-1^ |
| Phospholipid | 1.006 [0.925 1.143] | 1.035 [0.934 1.099] | -0.02 | 9.31x10^-1^ | 9.55x10^-1^ |
| Isobutyrate | 0.972 [0.895 1.035] | 0.918 [0.817 1.3] | -0.15 | 9.77x10^-1^ | 9.77x10^-1^ |

**Table S4.** Statistical comparison between the lipoprotein profiles of Metabotype II *versus* Metabotype III.

| **Feature** | **Metabotype II, median [IQR]** | **Metabotype III, median [IQR]** | **log change** | **p-value** | **FDR** |
| --- | --- | --- | --- | --- | --- |
| Main_Parameters_Triglycerides | 240.36 [214.002 299.433] | 91.02 [71.323 111.78] | 1.52 | 2.04x10^-6^ | 8.14x10^-5^ |
| MainFractions_Triglycerides_VLDL | 170.69 [159.183 210.132] | 49.29 [38.688 69.142] | 1.79 | 4.08x10^-6^ | 8.14x10^-5^ |
| MainFractions_Triglycerides_IDL | 26.57 [25.95 36.155] | 5.215 [2.48 8.28] | 2.47 | 4.08x10^-6^ | 8.14x10^-5^ |
| VLDL_Subfractions_Triglycerides_VLDL.1 | 107.94 [80.3 134.335] | 19.25 [11.785 27.652] | 2.45 | 4.08x10^-6^ | 8.14x10^-5^ |
| VLDL_Subfractions_Phospholipids_VLDL.1 | 15.77 [13.717 19.802] | 3.32 [2.45 4.475] | -2.2 | 4.08x10^-6^ | 8.14x10^-5^ |
| VLDL_Particle_Number | 278.955 [243.442 330.25] | 125.635 [95.94 150.235] | 1.26 | 7.14x10^-6^ | 8.14x10^-5^ |
| MainFractions_Cholesterol_VLDL | 43.075 [37.285 54.905] | 17.545 [11.707 21.458] | 1.42 | 7.14x10^-6^ | 8.14x10^-5^ |
| MainFractions_Cholesterol_IDL | 18.84 [15.553 28.68] | 9.94 [6.973 12.57] | 1.22 | 7.14x10^-6^ | 8.14x10^-5^ |
| MainFractions_Apo.B_VLDL | 15.34 [13.39 18.163] | 6.91 [5.275 8.262] | 1.26 | 7.14x10^-6^ | 8.14x10^-5^ |
| VLDL_Subfractions_Cholesterol_VLDL.1 | 20.4 [16.017 25.052] | 4.5 [2.473 6.945] | 2.2 | 7.14x10^-6^ | 8.14x10^-5^ |
| Main_Parameters_Apo.B100 | 105.6 [96.73 118.345] | 80.055 [67.373 86.227] | 0.45 | 3.06x10^-5^ | 2.49x10^-4^ |
| Total_Particle_Number | 1920.06 [1758.775 2151.845] | 1455.6 [1224.988 1567.833] | 0.45 | 3.06x10^-5^ | 2.49x10^-4^ |
| MainFractions_Phospholipids_VLDL | 43.66 [36.663 48.425] | 16.435 [12.775 21.26] | 1.35 | 3.06x10^-5^ | 2.49x10^-4^ |
| VLDL_Subfractions_Triglycerides_VLDL.2 | 23.955 [18.545 28.067] | 7.77 [6.612 12.015] | 1.31 | 3.06x10^-5^ | 2.49x10^-4^ |
| VLDL_Subfractions_FreeCholesterol_VLDL.3 | 3.745 [3.165 4.645] | 1.16 [0.662 1.658] | 1.65 | 4.59x10^-5^ | 3.27x10^-4^ |
| VLDL_Subfractions_Phospholipids_VLDL.2 | 6.555 [5.123 7.707] | 2.465 [1.86 3.462] | 1.22 | 4.59x10^-5^ | 3.27x10^-4^ |
| MainFractions_Phospholipids_IDL | 13.195 [11.277 13.99] | 4.77 [3.405 5.865] | 1.44 | 6.03x10^-5^ | 4.04x10^-4^ |
| VLDL_Subfractions_FreeCholesterol_VLDL.1 | 6.905 [6.43 8.595] | 1.345 [0.48 1.855] | 2.52 | 7.68x10^-5^ | 4.87x10^-4^ |
| MainFractions_FreeCholesterol_VLDL | 19.25 [17.398 23.538] | 7.74 [5.652 9.453] | 1.43 | 9.83x10^-5^ | 5.90x10^-4^ |
| MainFractions_FreeCholesterol_IDL | 5.695 [4.505 8.465] | 2.78 [1.777 3.518] | 1.32 | 1.40x10^-4^ | 6.73x10^-4^ |
| VLDL_Subfractions_FreeCholesterol_VLDL.2 | 3.625 [2.663 3.978] | 1.16 [0.845 1.642] | 1.55 | 1.41x10^-4^ | 6.73x10^-4^ |
| Main_Parameters_Cholesterol | 242.43 [216.257 251.153] | 186.395 [170.765 211.16] | 0.33 | 1.42x10^-4^ | 6.73x10^-4^ |
| VLDL_Subfractions_Cholesterol_VLDL.3 | 7.59 [6.305 9.242] | 2.66 [1.96 4.388] | 1.34 | 1.42x10^-4^ | 6.73x10^-4^ |
| VLDL_Subfractions_FreeCholesterol_VLDL.4 | 3.19 [2.522 5.088] | 1.405 [1.087 1.878] | 1.38 | 1.42x10^-4^ | 6.73x10^-4^ |
| VLDL_Subfractions_Triglycerides_VLDL.4 | 14.33 [12.16 18.005] | 7.765 [5.54 9.773] | 0.96 | 1.98x10^-4^ | 9.02x10^-4^ |
| VLDL_Subfractions_Phospholipids_VLDL.4 | 6.795 [5.648 8.938] | 3.99 [2.865 4.717] | 0.88 | 2.74x10^-4^ | 1.20x10^-3^ |
| IDL_Particle_Number | 123.81 [97.71 174.377] | 72.05 [59.055 92.15] | 0.93 | 3.73x10^-4^ | 1.32x10^-3^ |
| LDL.6_Particle_Number | 627.52 [479.947 741.995] | 346.56 [249.407 458.607] | 0.82 | 3.73x10^-4^ | 1.32x10^-3^ |
| MainFractions_Apo.B_IDL | 6.805 [5.375 9.592] | 3.965 [3.25 5.067] | 0.93 | 3.73x10^-4^ | 1.32x10^-3^ |
| LDL_Subfractions_Phospholipids_LDL.6 | 20.9 [18.147 23.673] | 14.04 [11.262 17.135] | 0.59 | 3.73x10^-4^ | 1.32x10^-3^ |
| LDL_Subfractions_Apo.B_LDL.6 | 34.515 [26.395 40.81] | 19.06 [13.715 25.225] | 0.82 | 3.73x10^-4^ | 1.32x10^-3^ |
| HDL_Subfractions_Triglycerides_HDL.4 | 4.755 [3.945 4.992] | 2.58 [2.433 2.852] | 0.77 | 3.73x10^-4^ | 1.32x10^-3^ |
| VLDL_Subfractions_Cholesterol_VLDL.2 | 7.385 [5.69 7.815] | 2.935 [2.002 3.798] | 1.24 | 3.95x10^-4^ | 1.32x10^-3^ |
| VLDL_Subfractions_Phospholipids_VLDL.3 | 7.77 [6.45 8.297] | 3.11 [2.045 3.857] | 1.21 | 3.95x10^-4^ | 1.32x10^-3^ |
| VLDL_Subfractions_Triglycerides_VLDL.3 | 21.9 [18.928 23.95] | 9.045 [5.5 11.13] | 1.15 | 4.41x10^-4^ | 1.44x10^-3^ |
| LDL_Subfractions_Cholesterol_LDL.6 | 39.615 [33.542 44.188] | 24.98 [18.985 31.68] | 0.68 | 5.04x10^-4^ | 1.60x10^-3^ |
| VLDL_Subfractions_Phospholipids_VLDL.5 | 2.29 [2.138 2.743] | 1.16 [0.915 1.9] | 0.87 | 6.12x10^-4^ | 1.89x10^-3^ |
| VLDL_Subfractions_FreeCholesterol_VLDL.5 | 1.66 [1.202 2.345] | 0.575 [0.302 0.908] | 1.49 | 6.70x10^-4^ | 2.01x10^-3^ |
| VLDL_Subfractions_Cholesterol_VLDL.4 | 7.425 [5.768 11.15] | 4.54 [3.668 5.143] | 0.9 | 8.84x10^-4^ | 2.58x10^-3^ |
| VLDL_Subfractions_Triglycerides_VLDL.5 | 3.69 [3.298 3.907] | 2.235 [1.852 2.918] | 0.6 | 1.15x10^-3^ | 3.28x10^-3^ |
| HDL_Subfractions_Triglycerides_HDL.3 | 2.645 [2.518 2.85] | 1.61 [1.395 1.738] | 0.71 | 3.84x10^-3^ | 1.07x10^-2^ |
| Apo.B100_Apo.A1 | 0.755 [0.705 0.815] | 0.59 [0.442 0.62] | 0.4 | 4.46x10^-3^ | 1.21x10^-2^ |
| MainFractions_Triglycerides_HDL | 12.12 [10.488 13.635] | 7.795 [6.595 9.035] | 0.63 | 5.91x10^-3^ | 1.57x10^-2^ |
| HDL_Subfractions_Apo.A2_HDL.3 | 6.88 [6.22 7.827] | 5.75 [5.462 6.303] | 0.3 | 9.97x10^-3^ | 2.58x10^-2^ |
| LDL_Subfractions_Triglycerides_LDL.6 | 6 [5.397 7.277] | 4.94 [4.215 5.12] | 0.34 | 1.08x10^-2^ | 2.74x10^-2^ |
| LDL_Subfractions_FreeCholesterol_LDL.6 | 8.035 [6.635 8.848] | 5.59 [4.115 7.095] | 0.54 | 1.18x10^-2^ | 2.92x10^-2^ |
| VLDL_Subfractions_Cholesterol_VLDL.5 | 1.88 [1.268 1.94] | 0.935 [0.682 1.532] | 0.73 | 1.28x10^-2^ | 3.10x10^-2^ |
| MainFractions_Apo.A2_HDL | 34.53 [34.012 39.838] | 31.145 [28.988 33.998] | 0.21 | 1.51x10^-2^ | 3.58x10^-2^ |
| HDL_Subfractions_FreeCholesterol_HDL.2 | 1.365 [1.005 1.562] | 1.705 [1.578 2.295] | -0.48 | 1.77x10^-2^ | 4.12x10^-2^ |
| LDL.Chol_HDL.Chol | 2.05 [1.872 2.608] | 1.66 [1.365 1.948] | 0.35 | 2.20x10^-2^ | 4.92x10^-2^ |
| HDL_Subfractions_Cholesterol_HDL.2 | 7.945 [6.66 8.887] | 10.025 [8.425 11.535] | -0.32 | 2.20x10^-2^ | 4.92x10^-2^ |
| Main_Parameters_Apo.A2 | 33.51 [31.752 39.38] | 30.085 [28.275 32.907] | 0.19 | 3.06x10^-2^ | 6.46x10^-2^ |
| LDL_Subfractions_Triglycerides_LDL.4 | 1.215 [1.105 1.64] | 2.055 [1.572 2.675] | -0.75 | 3.06x10^-2^ | 6.46x10^-2^ |
| LDL_Subfractions_FreeCholesterol_LDL.3 | 3.41 [1.525 3.945] | 4.775 [4.383 5.195] | -0.61 | 3.06x10^-2^ | 6.46x10^-2^ |
| Main_Parameters_HDL.Chol | 52.805 [51.48 56.24] | 60.32 [55.983 66.567] | -0.19 | 3.58x10^-2^ | 7.16x10^-2^ |
| MainFractions_Cholesterol_HDL | 52.805 [51.48 56.24] | 60.32 [55.983 66.567] | -0.19 | 3.58x10^-2^ | 7.16x10^-2^ |
| HDL_Subfractions_Apo.A1_HDL.3 | 25.605 [24.717 30.588] | 23 [22.125 27.023] | 0.17 | 3.58x10^-2^ | 7.16x10^-2^ |
| LDL.5_Particle_Number | 300.27 [247.382 321.38] | 166.685 [140.99 271.045] | 0.53 | 4.17x10^-2^ | 7.93x10^-2^ |
| LDL_Subfractions_Apo.B_LDL.5 | 16.515 [13.608 17.677] | 9.165 [7.755 14.91] | 0.53 | 4.17x10^-2^ | 7.93x10^-2^ |
| HDL_Subfractions_Apo.A2_HDL.4 | 21.8 [19.265 23.615] | 16.905 [14.2 20.055] | 0.27 | 4.17x10^-2^ | 7.93x10^-2^ |
| MainFractions_FreeCholesterol_HDL | 9.35 [9.21 10.613] | 11.74 [9.867 13.873] | -0.26 | 4.33x10^-2^ | 8.10x10^-2^ |
| LDL_Particle_Number | 1413.735 [1368.12 1703.253] | 1290.365 [1040.328 1393.905] | 0.27 | 4.84x10^-2^ | 8.76x10^-2^ |
| MainFractions_Apo.B_LDL | 77.755 [75.245 93.675] | 70.965 [57.218 76.665] | 0.27 | 4.84x10^-2^ | 8.76x10^-2^ |
| LDL_Subfractions_Cholesterol_LDL.5 | 22.305 [19.075 26.078] | 11.495 [8.82 21.198] | 0.57 | 6.43x10^-2^ | 1.11x10^-1^ |
| LDL_Subfractions_FreeCholesterol_LDL.2 | 4.665 [3.745 5.432] | 6.255 [4.715 6.837] | -0.34 | 6.43x10^-2^ | 1.11x10^-1^ |
| HDL_Subfractions_FreeCholesterol_HDL.1 | 3.275 [2.427 4.258] | 4.425 [3.698 5.373] | -0.43 | 6.43x10^-2^ | 1.11x10^-1^ |
| LDL_Subfractions_Triglycerides_LDL.1 | 6.215 [4.98 7.638] | 4.66 [3.415 6.215] | 0.41 | 8.41x10^-2^ | 1.39x10^-1^ |
| LDL_Subfractions_Phospholipids_LDL.2 | 8.03 [5.698 9.823] | 10.935 [8.47 11.943] | -0.36 | 8.41x10^-2^ | 1.39x10^-1^ |
| HDL_Subfractions_Apo.A1_HDL.1 | 21.315 [17.885 26.622] | 33.085 [21.492 38.812] | -0.39 | 8.41x10^-2^ | 1.39x10^-1^ |
| HDL_Subfractions_Apo.A2_HDL.2 | 4.045 [3.553 4.988] | 3.53 [2.595 4.11] | 0.37 | 9.50x10^-2^ | 1.54x10^-1^ |
| LDL_Subfractions_Phospholipids_LDL.5 | 11.475 [10.037 13.707] | 6.905 [5.468 11.435] | 0.45 | 9.56x10^-2^ | 1.54x10^-1^ |
| MainFractions_Phospholipids_HDL | 71.915 [67.455 74.508] | 78.99 [71.608 86.375] | -0.14 | 1.22x10^-1^ | 1.94x10^-1^ |
| MainFractions_Triglycerides_LDL | 21.375 [19.322 24.075] | 19.655 [15.92 20.8] | 0.16 | 1.38x10^-1^ | 2.15x10^-1^ |
| LDL_Subfractions_Triglycerides_LDL.2 | 1.91 [1.802 2.067] | 2.6 [1.895 3.11] | -0.41 | 1.43x10^-1^ | 2.18x10^-1^ |
| HDL_Subfractions_Triglycerides_HDL.2 | 2.015 [1.69 2.253] | 1.62 [1.118 1.89] | 0.37 | 1.43x10^-1^ | 2.18x10^-1^ |
| LDL_Subfractions_Cholesterol_LDL.2 | 15.645 [10.517 18.652] | 20.02 [14.985 21.427] | -0.28 | 1.54x10^-1^ | 2.25x10^-1^ |
| HDL_Subfractions_Phospholipid_HDL.1 | 18.775 [15.248 20.535] | 24.395 [19.26 30.763] | -0.3 | 1.54x10^-1^ | 2.25x10^-1^ |
| HDL_Subfractions_Phospholipid_HDL.2 | 13.235 [10.63 14.83] | 15.465 [12.505 17.177] | -0.17 | 1.54x10^-1^ | 2.25x10^-1^ |
| LDL_Subfractions_Triglycerides_LDL.5 | 3.56 [2.708 3.827] | 2.725 [2.22 3.428] | 0.25 | 1.69x10^-1^ | 2.43x10^-1^ |
| LDL_Subfractions_FreeCholesterol_LDL.5 | 5.4 [4.493 6.308] | 3.31 [2.975 5.9] | 0.28 | 1.88x10^-1^ | 2.66x10^-1^ |
| LDL_Subfractions_Phospholipids_LDL.3 | 6.72 [1.825 8.13] | 7.88 [5.605 8.828] | -0.45 | 1.92x10^-1^ | 2.66x10^-1^ |
| HDL_Subfractions_Cholesterol_HDL.1 | 16.89 [13.783 19.642] | 20.415 [15.768 26.862] | -0.28 | 1.92x10^-1^ | 2.66x10^-1^ |
| HDL_Subfractions_Apo.A1_HDL.4 | 73.3 [63.96 83.575] | 66.545 [59.858 75.243] | 0.13 | 2.35x10^-1^ | 3.23x10^-1^ |
| Main_Parameters_LDL.Chol | 109.14 [99.135 134.655] | 103.665 [83.118 113.803] | 0.19 | 2.59x10^-1^ | 3.39x10^-1^ |
| LDL.2_Particle_Number | 152.755 [114.168 179.565] | 187.735 [143.328 209.08] | -0.25 | 2.59x10^-1^ | 3.39x10^-1^ |
| MainFractions_Cholesterol_LDL | 109.14 [99.135 134.655] | 103.665 [83.118 113.803] | 0.19 | 2.59x10^-1^ | 3.39x10^-1^ |
| LDL_Subfractions_Apo.B_LDL.2 | 8.4 [6.28 9.872] | 10.325 [7.88 11.495] | -0.25 | 2.59x10^-1^ | 3.39x10^-1^ |
| Main_Parameters_Apo.A1 | 143.235 [136.41 150.05] | 138.3 [133.245 148.077] | 0.07 | 3.12x10^-1^ | 4.04x10^-1^ |
| HDL_Subfractions_Triglycerides_HDL.1 | 3.54 [2.73 3.958] | 2.96 [1.878 3.848] | 0.49 | 3.41x10^-1^ | 4.36x10^-1^ |
| HDL_Subfractions_FreeCholesterol_HDL.3 | 1.68 [1.388 1.87] | 1.76 [1.452 2.138] | -0.16 | 3.96x10^-1^ | 5.00x10^-1^ |
| LDL.3_Particle_Number | 131.705 [37.047 171.588] | 146.74 [108.203 174.567] | -0.36 | 4.03x10^-1^ | 5.00x10^-1^ |
| LDL_Subfractions_Apo.B_LDL.3 | 7.245 [2.04 9.44] | 8.07 [5.95 9.598] | -0.36 | 4.03x10^-1^ | 5.00x10^-1^ |
| LDL_Subfractions_Cholesterol_LDL.3 | 11.685 [2.105 15.377] | 13.41 [8.697 15.675] | -0.35 | 4.29x10^-1^ | 5.26x10^-1^ |
| HDL_Subfractions_Cholesterol_HDL.3 | 8.86 [8.398 10.175] | 9.345 [8.625 10.555] | -0.05 | 4.72x10^-1^ | 5.72x10^-1^ |
| LDL_Subfractions_Triglycerides_LDL.3 | 2.395 [1.852 2.637] | 2.475 [1.735 2.86] | -0.11 | 5.78x10^-1^ | 6.94x10^-1^ |
| MainFractions_Phospholipids_LDL | 59.635 [54.36 74.132] | 59.185 [49.73 65.748] | 0.09 | 5.85x10^-1^ | 6.95x10^-1^ |
| HDL_Subfractions_Phospholipid_HDL.3 | 15.095 [14.135 16.82] | 14.365 [13.732 17.263] | 0.01 | 6.25x10^-1^ | 7.35x10^-1^ |
| LDL_Subfractions_FreeCholesterol_LDL.4 | 3.065 [1.095 4.562] | 3.54 [2.478 4.383] | -0.14 | 6.82x10^-1^ | 7.93x10^-1^ |
| LDL_Subfractions_Cholesterol_LDL.1 | 18.885 [15.72 25.035] | 20.14 [13.995 26.125] | -0.03 | 7.52x10^-1^ | 8.66x10^-1^ |
| HDL_Subfractions_Apo.A1_HDL.2 | 17.805 [15.957 21.42] | 18.075 [15.985 20.025] | 0.06 | 8.38x10^-1^ | 9.43x10^-1^ |
| HDL_Subfractions_Phospholipid_HDL.4 | 26.26 [20.55 29.66] | 23.26 [21.895 27.84] | 0.06 | 8.41x10^-1^ | 9.43x10^-1^ |
| LDL.4_Particle_Number | 132.31 [49.392 180.19] | 126.205 [59.178 156.785] | 0.11 | 8.61x10^-1^ | 9.43x10^-1^ |
| LDL_Subfractions_Phospholipids_LDL.4 | 5.705 [1.485 8.697] | 6.23 [3.59 7.855] | -0.08 | 8.61x10^-1^ | 9.43x10^-1^ |
| LDL_Subfractions_Apo.B_LDL.4 | 7.275 [2.72 9.91] | 6.945 [3.255 8.625] | 0.11 | 8.61x10^-1^ | 9.43x10^-1^ |
| LDL.1_Particle_Number | 202.365 [170.42 230.515] | 209.91 [145.275 253.295] | -0.05 | 8.86x10^-1^ | 9.53x10^-1^ |
| LDL_Subfractions_Apo.B_LDL.1 | 11.13 [9.37 12.68] | 11.545 [7.992 13.93] | -0.05 | 8.86x10^-1^ | 9.53x10^-1^ |
| MainFractions_FreeCholesterol_LDL | 28.15 [25.31 36.675] | 29.46 [25.735 33.145] | 0 | 9.07x10^-1^ | 9.57x10^-1^ |
| HDL_Subfractions_Apo.A2_HDL.1 | 2.71 [2.225 3.487] | 2.98 [2.49 3.685] | 0.13 | 9.07x10^-1^ | 9.57x10^-1^ |
| MainFractions_Apo.A1_HDL | 140.92 [136.89 147.097] | 142.275 [135.855 148.532] | 0.01 | 9.31x10^-1^ | 9.65x10^-1^ |
| HDL_Subfractions_FreeCholesterol_HDL.4 | 3.055 [1.69 3.705] | 2.53 [2.2 3.5] | -0.01 | 9.31x10^-1^ | 9.65x10^-1^ |
| LDL_Subfractions_Cholesterol_LDL.4 | 10.105 [2.053 16.44] | 10.1 [5.402 13.578] | 0.04 | 9.53x10^-1^ | 9.70x10^-1^ |
| LDL_Subfractions_Phospholipids_LDL.1 | 10.87 [9.21 13.52] | 11.94 [8.565 14.76] | -0.09 | 9.53x10^-1^ | 9.70x10^-1^ |
| LDL_Subfractions_FreeCholesterol_LDL.1 | 5.19 [4.58 7.225] | 6.675 [4.457 7.775] | -0.09 | 9.77x10^-1^ | 9.77x10^-1^ |
| HDL_Subfractions_Cholesterol_HDL.4 | 19.02 [14.853 22.042] | 18.695 [16.23 22.68] | 0.01 | 9.77x10^-1^ | 9.77x10^-1^ |
